# l-Carnitine ameliorates congenital myopathy in a tropomyosin 3 de novo mutation transgenic zebrafish

**DOI:** 10.1186/s12929-020-00707-1

**Published:** 2021-01-12

**Authors:** Po-Jui Hsu, Horng-Dar Wang, Yung-Che Tseng, Shao-Wei Pan, Bonifasius Putera Sampurna, Yuh-Jyh Jong, Chiou-Hwa Yuh

**Affiliations:** 1 Institute of Molecular and Genomic Medicine, National Health Research Institutes, Zhunan, Miaoli Taiwan; 2 Institute of Biotechnology, National Tsing Hua University, Hsinchu, Taiwan; 3 Department of Laboratory Medicine, Mackay Memorial Hospital, Taipei, Taiwan; 4 Marine Research Station, Institute of Cellular and Organismic Biology, Academia Sinica, I-Lan County, Taiwan; 5 Department of Biological Science and Technology, National Chiao Tung University, Hsinchu, Taiwan; 6 Graduate Institute of Clinical Medicine, College of Medicine, Kaohsiung Medical University, Kaohsiung, Taiwan; 7 Departments of Pediatrics and Laboratory Medicine, Kaohsiung Medical University, Kaohsiung, Taiwan; 8 Translational Research Center of Neuromuscular Diseases, Kaohsiung Medical University, Kaohsiung, Taiwan; 9 Institute of Bioinformatics and Structural Biology, National Tsing Hua University, Hsinchu, Taiwan; 10 Ph.D. Program in Environmental and Occupational Medicine, Kaohsiung Medical University, Kaohsiung, Taiwan

**Keywords:** Congenital myopathy, Zebrafish, Tropomyosin 3 (TPM3), l-Carnitine

## Abstract

**Background:**

Congenital myopathy (CM) is a group of clinically and genetically heterogeneous muscle disorders, characterized by muscle weakness and hypotonia from birth. Currently, no definite treatment exists for CM. A de novo mutation in Tropomyosin 3-*TPM3*(E151G) was identified from a boy diagnosed with CM, previously *TPM3*(E151A) was reported to cause CM. However, the role of *TPM3*(E151G) in CM is unknown.

**Methods:**

Histopathological, swimming behavior, and muscle endurance were monitored in *TPM3* wild-type and mutant transgenic fish, modelling CM. Gene expression profiling of muscle of the transgenic fish were studied through RNAseq, and mitochondria respiration was investigated.

**Results:**

While *TPM3*(WT) and *TPM3*(E151A) fish show normal appearance, amazingly a few *TPM3*(E151G) fish display either no tail, a crooked body in both F0 and F1 adults. Using histochemical staining for the muscle biopsy, we found *TPM3*(E151G) displays congenital fiber type disproportion and *TPM3*(E151A) resembles nemaline myopathy. *TPM3*(E151G) transgenic fish dramatically swimming slower than those in *TPM3*(WT) and *TPM3*(E151A) fish measured by DanioVision and T-maze, and exhibit weaker muscle endurance by swimming tunnel instrument. Interestingly, l-carnitine treatment on *TPM3*(E151G) transgenic larvae significantly improves the muscle endurance by restoring the basal respiration and ATP levels in mitochondria. With RNAseq transcriptomic analysis of the expression profiling from the muscle specimens, it surprisingly discloses large downregulation of genes involved in pathways of sodium, potassium, and calcium channels, which can be rescued by l-carnitine treatment, fatty acid metabolism was differentially dysregulated in *TPM3*(E151G) fish and rescued by l-carnitine treatment.

**Conclusions:**

These results demonstrate that *TPM3*(E151G) and *TPM3*(E151A) exhibit different pathogenicity, also have distinct gene regulatory profiles but the ion channels were downregulated in both mutants, and provides a potential mechanism of action of TPM3 pathophysiology. Our results shed a new light in the future development of potential treatment for *TPM3*-related CM.

## Background

Congenital myopathy (CM) is a group of genetic muscle disorders characterized clinically by generalized hypotonia and muscle weakness present at birth and pathologically by the presence of specific morphological features on muscle biopsy, with estimated prevalence about 1:25,000 [1]. According to the main muscle pathological features, the most common types of CM are cores (central core disease), central nuclei (myotubular and centronuclear myopathy), rods (nemaline myopathy), and selective hypotrophy of type 1 fiber (congenital fiber type disproportion, CFTD). In striated muscles, tropomyosin interacts with the troponin complex to regulate actin-myosin interaction. Mutations of tropomyosin 3 (*TPM3*) cause CM with nemaline myopathy, cap myopathy, and CFTD [2, 3]. *TPM3*-related CM were reported to exhibit lowered Ca^2+^-sensitivity and damaged acto-myosin cross-bridge cycling in slow fibers [4] and disturb regulatory function via altered actin affinity and tropomodulin binding [5]. To date, over 61 phenotypes and 44 genotypes of CM are reported [6]. Although, mutations in the different genes may cause the same phenotype and different mutations in the same gene can produce multiple different phenotypes. CM share five common key pathophysiological defects in sarcolemmal and intracellular membrane remodeling and excitation–contraction coupling, mitochondrial distribution and function, myofibrillar force generation, atrophy, and autophagy [7]. Currently, no known treatment exists for any type of CM, only supportive therapy. Using the power of the zebrafish model, researchers are able to establish novel insights into pathomechanisms of CM and provide the necessary groundwork for future therapy development [8].

l-Carnitine is an amino acid that is widely present in human tissues and can be produced from essential amino acids lysine and methionine. It is responsible for sending fatty acids into the mitochondria to burn smoothly and produce energy. l-Carnitine significantly reduced statin-induced myopathy [9] and skeletal muscle atrophy in rats [10]. l-Carnitine improved exercise performance in human patients with mitochondrial myopathy [11]. l-Carnitine may prevent age-associated muscle protein degradation and regulate mitochondrial homeostasis [12]. Although dietary l-tyrosine supplementation was reported to improve bulbar function, activity levels, and exercise tolerance in some nemaline myopathy patients [13]. However, l-tyrosine supplementation was not able to attenuate the skeletal muscle dysfunction in zebrafish and the dominant skeletal muscle α-actin nemaline myopathy in mouse models [14].

A seven-year-old boy patient who had been suffering from generalized hypotonia, muscle weakness with myopathic face, high-arched palate, funnel chest, hyporeflexia, poor feeding, and motor delay since early infancy. He presented severe motor delay having head control at 8 months, was able to sit alone at 12 months, walk alone at 20 months, and still cannot run until seven years of age. As his parents refused the invasive clinical examination such as electromyogram or muscle biopsy for him, thus Dr. Yuh-Jyh Jong diagnosed with his serum DNA by using target gene capture/deep sequencing approach [15]. It revealed a novel and de novo mutation of *TPM3* at nucleotide 452 from A to G, resulting in amino acid 151 changing from glutamic acid (E) to glycine (G). Previously, a *TPM3* mutation changing nucleotide 452 from A to C resulting in amino acid 151 changing from glutamic acid (E) to alanine (A) was reported in a CM patient with cap myopathy [3]. To study the precise pathogenicity between *TPM3*(E151G) and CM, we generated transgenic zebrafish overexpressing the human wild type (WT) and two mutant *TPM3* genes in the muscle of zebrafish for functional analysis and potential drug screening. Here, we show that *TPM3*(E151G) transgenic zebrafish display much severe CM phenotypes than those from *TPM3*(E151A) and *TPM3*(WT) transgenic zebrafish. Remarkably, l-carnitine supplementation ameliorates the swimming speed and muscle endurance as well as rescues the downregulation genes involved in sodium, potassium, and calcium channels in *TPM3*(E151G) transgenic zebrafish by RNAseq. This study provides insights in the pathogenicity of the novel *TPM3*(E151G) mutation and a drug screening platform to uncover potential therapies for this rare disease.

## Materials and methods

### Zebrafish maintenance

All zebrafish were maintained at the Zebrafish Core Facility in National Health Research Institute (NHRI, Miaoli, Taiwan). Adult fish were maintained under an automated 14:10 h light/dark cycle and a constant temperature of 28 °C. All experiments involving zebrafish were approved by the Institution Animal Care and Use Committee (IACUC) of the NHRI (protocol No. NHRI-IACUC-103122-AE). The Taiwan Zebrafish Core Facility at NHRI or TZeNH is a government-funded core facility, and since 2015, it has been accredited by the Association for Assessment and Accreditation of Laboratory Animal Care International (AAALAC) [16].

### Transgenic zebrafish lines

AB wild-type was used in this study. Other transgenic fish lines, including Tg(MLC2:*TPM3*(WT);*myl7*:EGFP), Tg(MLC2:*TPM3*(E151A);*myl7*:EGFP), and Tg(MLC2:*TPM3*(E151G);*myl7*:EGFP) were generated in this study as described previously using Tol2 Gateway cloning Toolkit [17, 18]. Primers used for construct preparation are listed in Additional file 1: Table S1.

pME-*TPM3*(E151A) and pME-*TPM3*(E151G) were created by QuickChange II site-directed mutagenesis (Agilent Technologies, Santa Clara, CA, USA) from pME-*TPM3*(WT). All primers for mutagenesis are listed in Additional file 1: Table S1. The final expression construct pTol2-MLC2:*TPM3*(E151A):pA/CG2 was generated using Gateway® LR reaction (Invitrogen, Waltham, Massachusetts, USA) using p5E-MLC2, pME-*TPM3*(E151A), p3E-polyA, and pDestTol2CG2 vectors. The final expression construct pTol2-MLC2:*TPM3*(E151G):pA/CG2 was generated by Gateway® LR reaction (Invitrogen, Waltham, Massachusetts, USA) using p5E-MLC2, pME-*TPM3*(E151G), p3E-polyA, and pDestTol2CG2 vectors.

Three constructs were created by Tol2-Gateway cloning, and confirmed by real-time reverse transcription-PCR (qRT-PCR) and sequencing, and microinjected into AB wildtype zebrafish embryos. Expressions of transgenes were using by heart specific promoter myl7-driven green florescence protein (GFP) expression in 48 hours-post-fertilization (hpf) embryos and sequencing confirmed with genomic DNA extracted from adult fin. The mRNA level of *TPM3* transgenic zebrafish were examined by qRT-PCR, which confirmed all three *TPM3* transgenic zebrafish expressed human *TPM3* gene.

### Embryos collection

Embryos were collected once per week with the best results produced with a regular schedule. The night before embryo collection, adult male and female zebrafish were placed into a mating tank with a clapboard after the last feeding. Next morning, the clapboard was removed to start mating. Embryos were collected after 1 hour, and we removed the dead and unfertilized embryos. We transferred healthy embryos to a clean petri dish containing E3 medium (5 mM NaCl, 0.17 mM KCl, 0.33 mM CaCl_2_, and 0.33 mM MgSO_4_). We maintained the embryos in an incubator at 28 °C.

### Microinjection and selection and confirmation of transgenic zebrafish

We inserted a needle into the Nanoject II™ Nanoliter injector (Drummond Scientific, Broomall, PA, USA). For DNA and RNA co-injection, we injected 2.3 nL containing 0.05% phenol red into an embryo at the one-cell stage. We ensured the embryos did not develop past the two-cell stage. After injection, embryos were placed in E3 medium and incubated at 28 °C.

Two days after microinjection, we used a fluorescent microscope to screen the hearts of zebrafish embryo for green fluorescence. When the transgenic zebrafish were 3 months old, DNA was extracted by cutting the fins. After amplification of the target fragment by PCR, we entrusted the DNA Sequencing Core Lab at the National Health Research Institutes to sequence and confirm the target sequence.

### Fish length and body weight measurement and morphological analysis

Before examination, the fish was anesthesia using 0.0125% buffered MS-222 solution (ethyl 3-aminobenzoate methane sulfonic acid salt, Sigma-Aldrich, St. Louis, MI, USA). Total length and standard length were measured using Vernier calipers with 0.1 mm accuracy. Fish body weight was determined to an accuracy of 0.01 g. For morphological analysis, a digital CMOS X-ray detector (Model 2315, Dexela, London, UK), set to 45 kV and 120 mA, with 2.5 seconds of exposure, was used to view vertebrae of the fish [19].

### Tracking of larvae fish swimming behavior

The zebrafish larvae swimming behavior data were tracked and acquired by using DanioVision equipment (Noldus, Wageningen, The Netherlands). Before the indicated times of development, zebrafish larvae were placed individually into a 48-well plate with 1200 µL E3 medium overnight. According to DanioVision manual, we used light-induced visual motor response analysis. The swimming behavior test for zebrafish larvae involved the following conditions: the first 30 min was required for their adaptation, then we used a light switch (20 min: 10 min in dark and 10 min in light) for 2 cycles [20, 21]. The water temperature during the experiment was maintained at 28 ± 0.5 °C.

### T-maze behavior test

A three armed T-maze was used for our experiment. The stem of the maze (length 36 cm × width 11 cm × height 20 cm) included the start box (11 × 11 × 20 cm) and each arm of the maze (25 × 11 × 20 cm). Blue or red cellophane was attached to the end of the arm. The T-maze was filled with system water and the temperature was maintained at 28 ± 1 °C.

For the zebrafish adult behavior test, we used T-maze for memory testing and velocity testing. The T-maze behavior test is divided into three phases: pre-training, training, and testing. In the pre-training phase, adult zebrafish were placed in a 3 L fish tank when they were 3 months old with a white sponge filled with food to induce the fish to find the white sponge twice each day (10:00 and 15:00) for one week. In the training phase, adult zebrafish were moved to the behavioral room and placed into an individual tank. All fish were acclimatized for 1 h before placement into the T-maze. During each trial, a zebrafish was placed in the start box for 1 min with the door closed. Then, the door was raised and closed after the fish had left. A stopwatch was used to measure the time required for the fish to find the white sponge filled with food. If the zebrafish did not leave the start box or find the target within 3 min, the experiment was terminated. In training phase, each fish was trained once each day for one week. In the testing phase, adult zebrafish were moved to the behavioral room and placed into an individual tank. All fishes acclimatized for 1 h before placement into the T-maze. During each trial, a zebrafish was placed in the start box for 1 min with the door closed. Then, the door was raised and closed after the fish had left. A stopwatch was used to measure the time required for the fish to find the white sponge. If the zebrafish did not leave the start box or find the target within 3 min, the experiment was terminated. In testing phase, each fish was tested once per day for one week.

### Analysis of adult fish T-maze behavior and larvae fish swimming behavior test

The zebrafish adult behavior test and larvae swimming behavior test were analyzed using EthoVision XT 13 (Noldus, Wageningen, the Netherlands). We evaluated the velocity, latency, and distance movement of individual zebrafish.

### Adult fish muscle endurance swimming performance

Critical swimming speed *(U*_crit_*)* is defined as the maximum speed an adult fish can sustain over a period of time [22]. Critical swimming speed was measured in Brett-type swimming tunnel (Loligo Systems, Viborg, Denmark). Fish were not fed for 24 hours before measurements. One fish was selected using a net from the fish tank and placed in the swimming tunnel. After the resting habituation for 5 min, water velocity was gradually increased by 10 cm/s in intervals of 5 min until the fish apparaently showed exhausted swimming performance. The water temperature during experiments was 28 ± 1 °C. *U*_crit_ was calculated using [22].

$$U_{{{\text{crit}}}} = U_{i} + [U_{ii} \left( {T_{i} /T_{ii} } \right)]$$

where *U*_i_ is the highest velocity the fish maintained for the whole 5 min (cm/s), *U*_ii_ is the velocity increase, *T*_i_ is the time elapsed at the tired velocity, and *T*_ii_ is the time between velocity changes (5 min).

### Analysis of adult fish muscle endurance swimming performance

Principal component analysis (PCA) was performed using XLSTAT 2014.1 (Addinsoft, NY, USA) to identify the main cause of induced responses and the relationship between these parameters. A biplot was graphed of both the measured parameters and observations.

### Chemical treatment for larval fish

Six chemicals, including l-tyrosine, Taurine, l-carnitine, creatine, Terazosin and ATP, were used to test their effects. Four chemicals: l-tyrosine (All Lines Technology, PA, USA), Taurine (NIPPON SHINYAKU, Kyoto, Japan), l-carnitine (Sigma-Aldrich, MI, USA) and creatine (Sigma-Aldrich, MI, USA) were dissolved in water at a concentration of 10 µM, 1 mM, 10 µM and 100 µM respectively. The concentration were determined according to a previous report on dosage analysis [23]. Terazosin (Selleckchem, TX, USA) was dissolved in Dimethyl sulfoxide (DMSO) at a concentration of 2.5 µM following a previous report on dosage analysis [24]. ATP (Sigma-Aldrich, MI, USA) was dissolved in water at a concentration of 100 µM. For treatment of the *TPM3* transgenic zebrafish model, *TPM3* transgenic adult F2 zebrafish were in-crossed to produce the F3 embryos. Following a previous method [23], embryos were immersed with different chemicals all day from 28 hours-post-fertilization (hpf) to 7 days-post-fertilization (dpf) in a 90 mm dish. The chemical was refreshed every day until 7 dpf. Then, zebrafish larvae were transferred into 48-well plates with 1200 µL E3 medium for the larvae fish swimming behavior test using DanioVision.

### L-Carnitine treatment of larvae fish for one month

The larva treated l-carnitine from 28 hpf to 7 dpf in 90 mm dish were continuous treatment from 7 to 30 dpf by immersed in l-carnitine from 20:00 to 08:00 in a 1 L fish tank. The body weight and body length after treatment were measured for all fish. The swimming tunnel (Loligo Systems, Viborg, Denmark) were used to measure critical swimming speed for the restoration effect of swimming behavior of *TPM3* transgenic zebrafish after l-carnitine treatment.

### L-carnitine treatment of adult fish from 3 months of age

*TPM3* transgenic zebrafish were immersed in 10 µM l-carnitine from 3 months of age from 20:00 to 08:00 for one month. The body weight and body length were measured after treatment. Swimming tunnel (Loligo Systems, Viborg, Denmark) were used to measure the critical swimming speed for the rescue effect of l-carnitine treatment.

### Oxygen consumption rate for determination of respiratory function

Zebrafish embryos develop until 48 hpf. Oxygen consumption rate (OCR) was measured from embryos using a V17 Islet capture plate in the Seahorse XFe24 Analyzer (Agilent, CA, USA). E3 medium was used in wells, and two dechorionated embryos were placed in each well. All measurement cycles for the run were set to 3 min mix, 2 min wait, and 3 min measure. Oligomycin was then added followed by 3 measurement cycles. Carbonyl cyanide 4-(trifluoromethoxy)phenylhydrazone (FCCP) was then added followed by another 3 measurement cycles. Finally, a mixture of antimycin and rotenone was added followed by 10 measurement cycles. Optimal doses of drugs were optimized separately for *TPM3*(WT) and *TPM3* mutant embryos. Final concentrations of drugs were as follows: *TPM3*(WT) oligomycin 50 μM, *TPM3* mutant oligomycin 25 μM, *TPM3*(WT) FCCP 20 μM, and *TPM3* mutant FCCP 3 μM. Antimycin and rotenone were added to a final concentration of 2 μM for both.

### Tissue collection, frozen section, and histological analysis

Before examination, the fish was anesthetized by 0.025% buffered MS-222 solution (Sigma-Aldrich, MI, USA) following a previously reported method [25]. We cut the muscle tissue between the anal and caudal fins and placed the muscle tissue in wet gauze. We placed 10% Tragacanth gum (FUJIFILM Wako Pure Chemical Corporation, Tokyo, Japan) on the well, which was stirred evenly, and formed into a small hill shape. We placed the muscle tissue into the small hill. Then, a beaker filled with 100 mL 2-methylbutane (Sigma-Aldrich, MI, USA) and placed into liquid nitrogen. We completely submerged the wood with muscle tissue into 2-methylbutane for 1 min. We then transferred muscle tissue to a − 80 °C freezer immediately. The muscle tissue was sectioned into 8 µm slices using Cryostat Microtome CM3050S (Leica, Wetzlar, Germany). Tissue sections were stored in a − 80 °C freezer, and later stained with Hematoxylin and Eosin stain (HE stain), Gomori’s trichrome stain, and nicotinamide adenine dinucleotide dehydrogenase-tetrazolium reductase stain (NADH-TR stain).

### Total RNA isolation, qRT-PCR (real-time reverse transcription-PCR)

The muscle tissue was grounded using Microtube Pellet Pestle Rods (Violet BioScience, Taipei, Taiwan). TRI Reagent® (Sigma-Aldrich, MI, USA) was mixed with sample and homogenized using a homogenizer. 1/5 of chloroform was added, mixed, and later the supernatant containing RNA was transferred to a new Eppendorf. After extracting total RNA from muscle tissue, the total RNA was applied in NucleoSpin® RNA kit (Macherey–Nagel, Nordrhein-Westfalen, Germany) for the cleanup. RNA was isolated using a NucleoSpin® RNA column (Macherey–Nagel, Nordrhein-Westfalen, Germany) per manufacturer‘s protocol. Finally, for all the RNA samples, we measured the concentration using a NanoDrop ND-1000 (Thermo Fisher Scientific, MA, USA), which were then stored in a − 80 °C freezer. Complementary DNA (cDNA) was synthesized using iScript™ cDNA synthesis kit (BioRad, CA, USA). cDNA was diluted 100-fold for qRT-PCR analysis using SYBR Green (Thermo Fisher Scientific, MA, USA). Primers for qRT-PCR are listed in Additional file 1: Table S1.

### Next generation sequencing analysis

Next generation sequencing experiment was performed by NHRI Genomics Core Facility. Sequencing libraries 1 µg total RNA were generated using RNA HyperPrep kit with RiboErase (Kapa Biosystems, Pleasanton, CA, USA), and sequenced on NovaSeq 6000 instrument (Illumina, San Diego, CA, USA). All RNA sample preparation procedures were carried out according to the Illumina's official protocol. Agilent's SureSelect Strand-Specific RNA Library Preparation Kit was used for library construction followed by AMPure XP beads (Beckman Coulter, USA) size selection. Illumina's bcl2fastq program v2.20 used for basecalling. De-multiplexed sequencing data was processed with Trimmomatic (version 0.36) to remove any adapter and poor-quality sequences with a sliding-window approach. Resulting read pairs were aligned with a HISAT2 aligner against zebrafish GRCz11 reference genomes (Ensembl version 99) to obtain reads count and fragments per kilobase per million (FPKM) data of genes and transcripts.

The expression analysis settings were as follows: Gene-level fold change <  − 2 or > 2, gene-level *p*-value < 0.05, ANOVA method: Ebayes. The gene ontology analysis was performed using gene ontology analysis via WEB-based GEne SeT AnaLysis Toolkit (WebGestalt [26], http://www.webgestalt.org/), pathway analyses were performed by NetworkAnalyst (http://www.networkanalyst.ca/), and activated pathways were selected and matched according to the Kyoto Encyclopedia of Genes and Genomes (KEGG) database.

### Statistical analysis

Data were collected and analyzed using Excel (Microsoft, WA, USA) and GraphPad Prism 9 software (GraphPad Software, CA, USA). Individual tests were described in the main text. All the statistical analyses were conducted using two-tailed Student’s *t*-test. All data are expressed as mean ± standard error of the mean (SEM). A *P*-value less than 0.05 was considered statistically significant. For all figures: NS, *P* > 0.05; *, *P* ≤ 0.05; **, *P* ≤ 0.01; ***, *P* ≤ 0.001; ****, *P* ≤ 0.0001.

## Results

### *TPM3* mutant transgenic fish shows abnormal appearance and lower survival rate

**Fig. 1:**
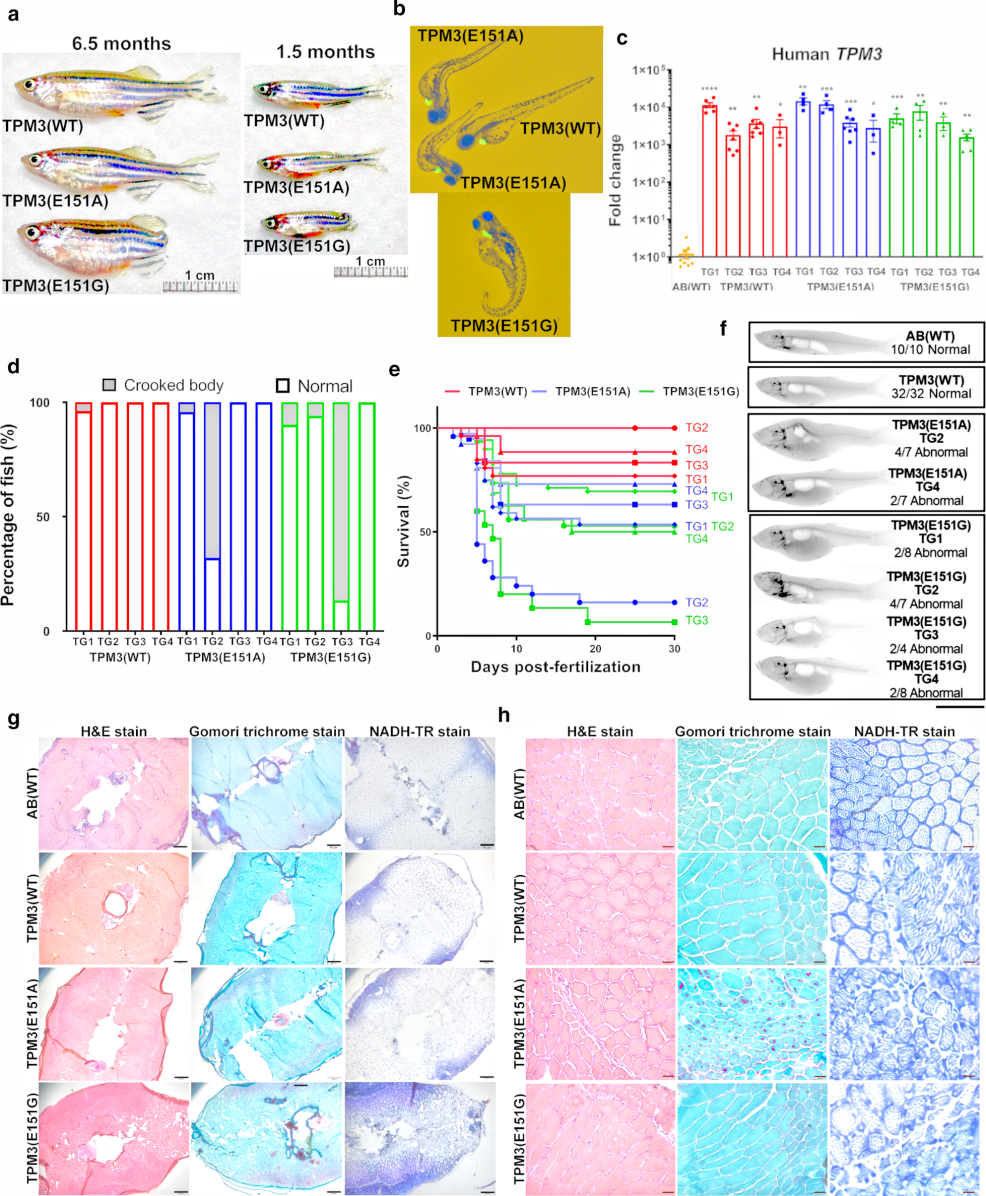
Appearance, survival rate, X-ray images and histopathological examinations of muscle in *TPM3* transgenic zebrafish. **a** Appearance of F0 adult *TPM3* transgenic fish at 6.5 months of age and F1 adult *TPM3* transgenic fish at 1.5 months of age. Scale bar is 1 cm. **b** Representative images of the F1 larvae *TPM3* transgenic fish. **c** Expression level of human *TPM3* mRNA in *TPM3* transgenic fish compared to non-transgenic control AB(WT). Orange bar represents AB(WT), red bar denotes four TG lines of *TPM3*(WT), blue color indicates four TG line of *TPM3*(E151A), green plot indicates four TG lines of *TPM3*(E151G). **d** Phenotypical analysis of the F1 larvae *TPM3* transgenic fish, crooked body were indicated as grey color, *TPM3* mutant lines exhibited higher percentage of abnormaility compared to *TPM3*(WT) **e** Survival rate of the F1 larva of *TPM3* transgenic fish, *TPM3* mutant lines exhibited lower survival rate compared to *TPM3*(WT). **f** X-ray images of skeleton alignment for adult AB(WT), *TPM3*(WT), *TPM3*(E151A), and *TPM3*(E151G). Zebrafish have 31 vertebrae in male and female fish. AB(WT) and *TPM3*(WT) exhibited normal skeleton alignment. *TPM3*(E151A) TG2 and TG4 showed abnormal skeleton alignment. All four independent lines of *TPM3*(E151G) transgenic zebrafish displayed some levels of abnormal skeleton alignment. Scale bar is 1 cm. **g** Hematoxylin and Eosin stain (HE) stain, modified Gomori trichrome stain, and NADH-TR stains of adult AB(WT) and adult F1 *TPM3* transgenic zebrafish at 50X, scale bar is 200 µm. **h** Representative images of three staining at 400X, scale bar is 50 µm

Three transgenic fish expressing human *TPM3*(WT), *TPM3*(E151A), and *TPM3*(E151G) under the promoter of myosin light chain 2 (*MLC2*) were generated as described previously [17] with details in materials and methods. The mRNA level of *TPM3* transgenic zebrafish were examined by qRT-PCR and confirmed all three *TPM3* transgenic zebrafish expressed human *TPM3* gene (Additional file 2: Fig. S1A). When compared to the non-transgenic AB(WT) control fish, overexpressed *TPM3*(WT) or *TPM3*(E151A) mutant, those fish displayed normal appearance, however, overexpressed E151G mutant *TPM3*, the fish showed abnormal appearance with no tail as early as F0 (Fig. 1a). This abnormal morphology was inherited to F1 adult fish (Fig. 1a). The F1 larvae from *TPM3*(E151G) and *TPM3*(E151A) transgenic fish also exhibited a higher proportion of abnormalities (Fig. 1b). Since this de novo mutation is heterogeneous in patient, it is genetic dominant. To clarify whether the observed defect is really due to the expression of the *TPM3* gene rather than other nonspecific effects, we have measured the human *TPM3* RNA expression via qRT-PCR analysis using RNAs from various transgenic strains and showed the transgenic fish indeed overexpression human *TPM3* gene (Fig. 1c).

Among four independent lines of *TPM3*(E151G) fish, 9.8% of TG1, 5.9% of TG2, and 86.7% of TG3 displayed a crooked body appearance. Even for the *TPM3*(E151A) fish, 4.2% of TG1 and 68% of TG2 had crooked body appearance (Fig. 1d). The survival rate of F1 larvae was affected by the *TPM3* mutations. In general, the survival rates of both *TPM3* mutants were decreased compared to *TPM3*(WT), and *TPM3*(E151G) had a much lower survival rate than *TPM3*(E151A) (Fig. 1e).

### *TPM3*(E151G) exhibits more severe vertebrae abnormalities than *TPM3*(E151A)

As the mutant *TPM3* transgenic zebrafish showed abnormal body appearances, we further examined the skeleton imaging of the *TPM3* transgenic zebrafish using a digital X-ray detector [19]. The non-transgenic AB(WT) control fish has compared to the wildtype or mutant *TPM3* transgenic fish for the skeleton, ten AB(WT) fish were all normal (Fig. 1f). Four independent lines of *TPM3*(WT) F1 adult fish all had normal aligned vertebrae (Fig. 1f). In the two independent TG2 and TG4 lines of *TPM3*(E151A) F1 fish, we observed four out of seven fish (57.1%) and two out of seven fish (28.5%) respectively with malformations in the vertebrae (Fig. 1f). All four independent lines of *TPM3*(E151G) F1 adult fish exhibited skeleton abnormalities, with malformations in two out of eight (25%), four out of seven (57.1%), two out of four (50%), and two out of eight fish (25%) for TG1 to TG4, respectively (Fig. 1f). The X-ray imaging results showed that overexpression of wildtype *TPM3* does not contribute to pathology, *TPM3* mutations produce a deformity in the precaudal vertebrae in most fish, and that in *TPM3*(E151G) was more severe than in *TPM3*(E151A).

### *TPM3*(E151G) displays congenital fiber type disproportion and *TPM3*(E151A) resembles nemaline myopathy

Muscle biopsy is an important diagnostic resource for confirming CM. We used the cryosection and histochemical staining to examine the muscle specimens of *TPM3* transgenic zebrafish in F1 adults, the non-transgenic AB(WT) control has compared to *TPM3* transgenic fish for the muscle pathology. Both AB(WT) and *TPM3*(WT) fish all had normal muscle cell from all three staining. By HE stain, we observed that the muscle fibers of *TPM3*(WT) were arranged neatly and consistent, *TPM3*(E151A) fish shows smaller-sized fibers and *TPM3*(E151G) shows variation in fiber size (Fig. 1g, h). *TPM3*(E151A) fish had rod-like structures in the muscle fibers resembled nemaline myopathy on Gomori trichrome stain (Fig. 1g, h). In zebrafish, type 1 fiber is usually smaller than type 2 fibers. Using NADH-TR stain under low magnification (50X), the *TPM3*(WT) fish have both type 1 (darker) and type 2 (paler) fibers similar to non-transgenic control AB(WT) (Fig. 1g). *TPM3*(E151A) transgenic fish also showed both types. On the other hand, *TPM3*(E151G) has increased smaller-sized and darkly stained fibers (Fig. 1g), the muscle fibers of *TPM3*(E151G) displayed small and dark-stained type 1 muscle fibers and disproportionate arrangement, similar to CFTD (Fig. 1g, h).

To further elucidate whether expression of WT or mutant *TPM3* caused fiber type conversion, we performed qRT-PCR for the expression level of type 1 and type 2 heavy chain. The myosin heavy chain isoform for type 1 muscle is encoded by *myh7*, and type 2A is encoded by *myhz2* in zebrafish [27], we measured the expression of *myh7* and *myhz2* for the AB(WT), wildtype and mutant *TPM3* transgenic fish. Both *TPM3*(WT) and *TPM3*(E151A) expressed higher level of type 1, and lesser type 2 myosin heavy chain compared to AB(WT), so there is fiber type conversion. On the other hand, *TPM3*(E151G) expressed lower levels of both type 1 and type 2 myosin heavy chain (Additional file 2: Fig. S1A, B). So, it seems like there is no fiber type conversion in *TPM3*(E151G), but generally losing both muscle type.

### *TPM3* mutant transgenic fish display reduced swimming ability compared to *TPM3*(WT) fish

**Fig. 2:**
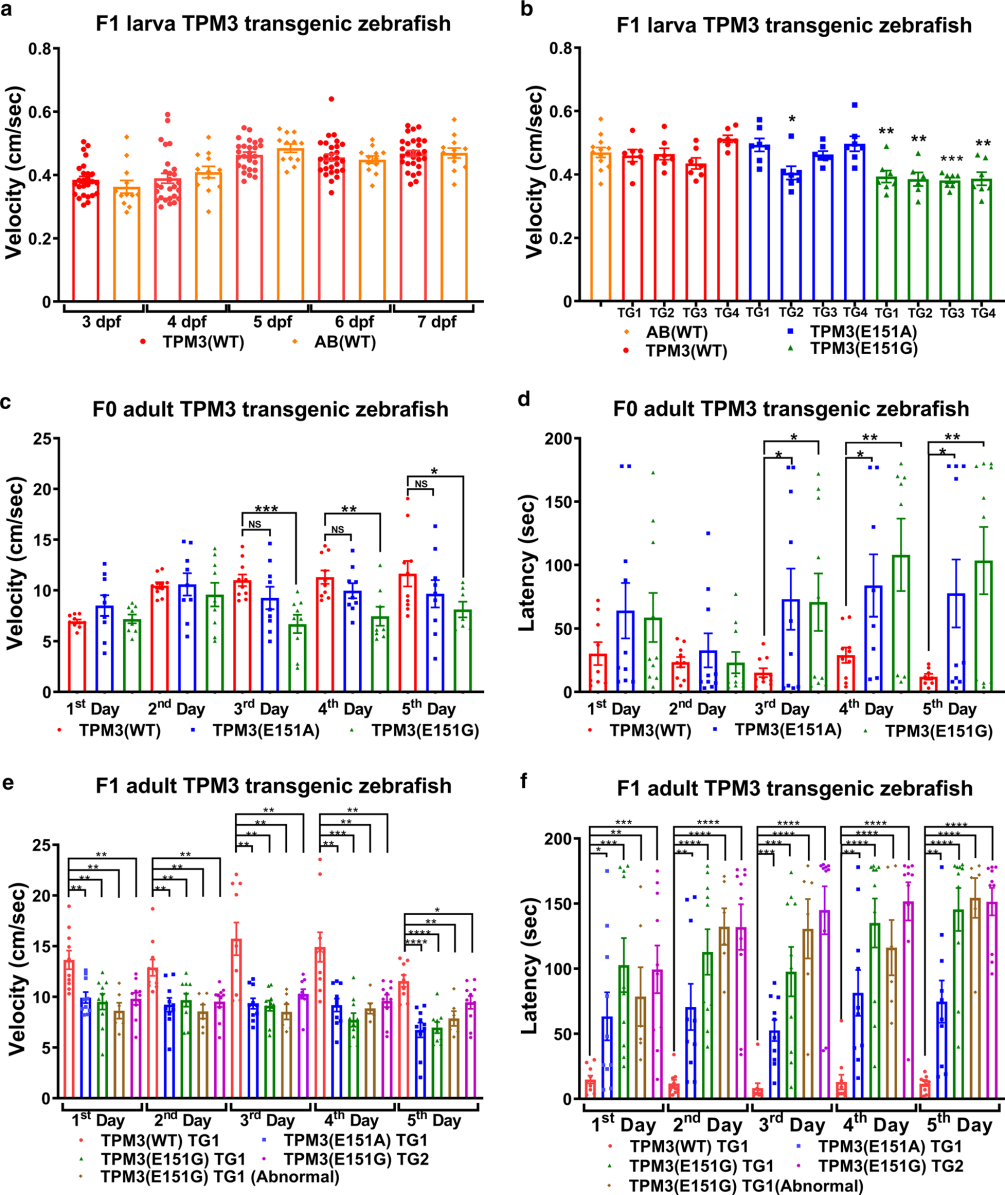
T-maze behavior test for adult *TPM3* transgenic zebrafish, and the swimming velocity of larval *TPM3* transgenic zebrafish. **a** The swimming velocity of F1 larva *TPM3*(WT) transgenic fish compared to non-transgenic AB(WT) control fish during the five days of testing. Red plot denotes *TPM3*(WT) fish (*n* = 28), orange plot represents AB(WT) fish (*n* = 12). There is no significant differences between *TPM3*(WT) and AB(WT). **b** The swimming velocity of F1 larva *TPM3* transgenic fish at 7 dpf. Orange plot represents AB(WT) (*n* = 12), red plot denotes four TG lines of *TPM3*(WT) (*n* = 8 for each line), blue plot indicates four TG line of *TPM3*(E151A) (*n* = 8 for each line), green plot indicates four TG lines of *TPM3*(E151G) (*n* = 8 for each line). Four lines of *TPM3*(E151G) and TG2 of *TPM3*(E151A) significantly swim slower compared to AB(WT). **c** The swimming velocity of F0 adult *TPM3* transgenic zebrafish during the five days of testing. Red plot represents *TPM3*(WT) (*n* = 10), blue plot indicates *TPM3*(E151A) (*n* = 10), green plot indicates *TPM3*(E151G) (*n* = 10). **d** Latency for memory test of F0 adult *TPM3* transgenic zebrafish during the five days of testing. *TPM3*(WT), *TPM3*(E151A), and *TPM3*(E151G) are shown in red, blue, and green bars, respectively. **e** The swimming velocity of F1 adult *TPM3* transgenic zebrafish during the five days of testing. Red plot represents *TPM3*(WT) TG1 (*n* = 10), blue plot denotes *TPM3*(E151A) TG1 *(n* = 10), green plot indicates *TPM3*(E151G) TG1 (*n* = 10), brown plot is *TPM3*(E151G) TG1 abnormal appearance (*n* = 6), and pink plot is *TPM3*(E151G) TG2 (*n* = 10). **f** Latency for memory test of F1 adult *TPM3* transgenic zebrafish during the five days of testing. *TPM3*(WT) TG1, *TPM3*(E151A) TG1, *TPM3*(E151G) TG1, *TPM3*(E151G) TG1 abnormal appearance, and *TPM3*(E151G) TG2 are shown in red, blue, green, brown, and pink bars, respectively. Statistical significance was determined in comparison with AB(WT) and was determined using a *t*-test, *0.01 < *P* ≤ 0.05; **0.001 < *P* ≤ 0.01; ***0.0001 < *P* ≤ 0.001; ****P ≤ 0.0001

To examine whether the affected muscle development in *TPM3* mutants may lead to swimming disability, the swimming velocity and latency of *TPM3* transgenic fish were measured by using DanioVision for larva and T-maze for adult zebrafish. The AB(WT) fish control were compared to the wildtype or mutant *TPM3* transgenic fish for the swimming velocity, and there is no difference between AB(WT) and wildtype *TPM3* transgenic fish larva (Fig. 2a); the four independent lines of *TPM3*(E151G) at 7 dpf all exhibited significant lower velocity compared to AB(WT) fish (Fig. 2b). *TPM3*(E151A) TG2 swimming velocity was also significantly lower compared with the *TPM3*(WT) fish at 7 dpf (Fig. 2b). In addition, we measured the swimming velocity of 3 to 6 dpf, and found that *TPM3*(E151G) larvae exhibited lower velocity compared to AB(WT) (Additional file 2: Fig. S1C-F). Whereas the F0 *TPM3*(E151G) adult fish after day 3 to day 5 exhibited significantly lower swimming velocity compared to *TPM3*(WT), we found no difference between *TPM3*(WT) and *TPM3*(E151A) (Fig. 2c). However, regarding the latency, *TPM3*(E151G) and *TPM3*(E151A) required significantly longer time to find the target compared with *TPM3*(WT) after day 3 to day 5 (Fig. 2d). We also examined those in the F1 adult of the *TPM3* transgenic fish, and found that both *TPM3*(E151A) and *TPM3*(E151G) F1 adults still exhibited significantly lower swim velocity compared with *TPM3*(WT) F1 adult (Fig. 2e). We also found the two groups of *TPM3*(E151G) TG1 line, with or without tails (abnormal), all swam slower than *TPM3*(WT). In latency, all F1 adult fish of the *TPM3*(E151G) and *TPM3*(E151A) required significantly longer time to find the target compared with *TPM3*(WT) F1 adult (Fig. 2f). The data above indicate *TPM3* mutant transgenic fish display reduced swimming ability. The swimming behavior of *TPM3* transgenic fish are shown in Additional file 3: Movie S1.

### *TPM3* mutant transgenic fish discloses weaker muscle endurance

**Fig. 3:**
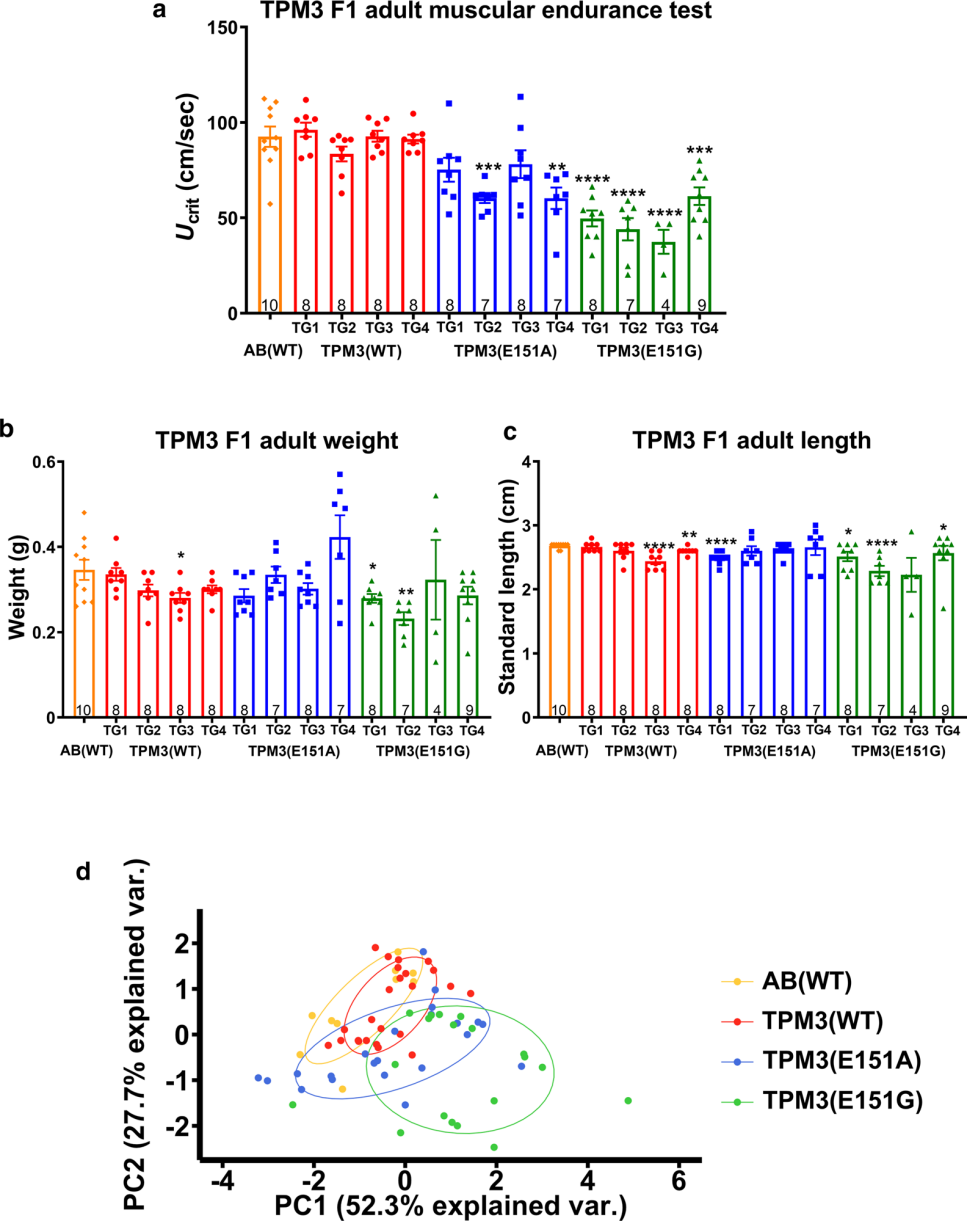
Muscle endurance test for adult F1 *TPM3* transgenic zebrafish. **a** Critical swimming speed (*U*_crit_) was used to measure the muscle endurance using a swimming tunnel. The statistical significance was calculated in comparison with AB(WT). The orange plot is AB(WT) (n = 10), red plot is the four TG lines of *TPM3*(WT) (TG1, *n* = 8; TG2, *n* = 8; TG3, *n* = 8; TG4, *n* = 8), blue plot is the four TG lines of *TPM3*(E151A) (TG1, *n* = 8; TG2, *n* = 7; TG3, *n* = 8; TG4, *n* = 7), green plot is the four TG lines of *TPM3*(E151G) (TG1, *n* = 8; TG2, *n* = 7; TG3, *n* = 4; TG4, *n* = 9). **b** The body weight of the 13 groups. **c** The standard length of the 13 groups. **d** Principal component analysis of endurance test for adult F1 *TPM3* transgenic zebrafish. The first principal component (PC) accounted for 52.3% and the second PC for 27.7% of the variance. The orange plot is AB(WT) (*n* = 10), red is *TPM3*(WT) (*n* = 24), blue is *TPM3*(E151A) (*n* = 22), and green is *TPM3*(E151G) (*n* = 19). Statistical significance was determined using a *t*-test, *0.01 < *P* ≤ 0.05; **0.001 < *P* ≤ 0.01; ***0.0001 < *P* ≤ 0.001; ****P ≤ 0.0001

The T-maze swimming test may combine learning and memory in the swimming behavior and may not be suitable to only represent the muscle strength. To precisely examine the strength of the muscles, critical swimming speed (*U*_crit_) to measure muscle endurance in a swimming tunnel for a total of 13 groups of F1 adult zebrafish including AB(WT) zebrafish, four independent lines for *TPM3*(WT), *TPM3*(E151A), and *TPM3*(E151G) was determined. A non-transgenic AB(WT) fish control has compared to the wildtype *TPM3* transgenic fish for the critical swimming speed, no differences in muscle endurance between AB(WT) and *TPM3*(WT) adult fish were detected (Fig. 3a). Importantly all four independent lines of *TPM3*(E151G) exhibited significantly debilitated muscle endurance compared with AB(WT) (Fig. 3a). Two independent lines TG2 and TG4 of *TPM3*(E151A) showed significantly weaker muscle endurance compared with AB(WT) (Fig. 3a). We showed zebrafish swimming near *U*_crit_ in the end of Additional file 3: Movie S1.

We wondered whether the body weight or length of these 13 groups might have resulted in the difference in muscle endurance test; only *TPM3*(WT) TG3, and *TPM3*(E151G) TG1 and TG2 had significantly lower body weight compared to AB(WT) (Fig. 3b). After standardizing the length, some of the fish, including *TPM3*(WT) TG3 and TG4, *TPM3*(E151A) TG1, and *TPM3*(E151G) TG1 and TG2, were significantly shorter, whereas *TPM3*(E151G) TG4 was significantly longer compared with AB(WT) (Fig. 3c). Therefore, there is no correlation between either body weight or length and muscle endurance.

Four variation factors, critical swimming speed (*U*_crit_), sex, body weight, and standard length, were analyzed by using principal component analysis. When two principle factors were selected, the total explanatory power was 80%. The first principal component (PC) and the second PC accounted for 52.3% and 27.7% of the variance, respectively. When the two PC factors represented all the factors, only 20% of the variation was ignored. We observed that AB(WT) and *TPM3*(WT) almost overlap, which we interpreted as the same group. *TPM3*(E151G) is separated from AB(WT) and *TPM3*(WT), which is obviously different from the AB(WT) and *TPM3*(WT) group (Fig. 3d).

### l-Carnitine specifically improves the swimming velocity of *TPM3*(E151G) larvae

**Fig. 4:**
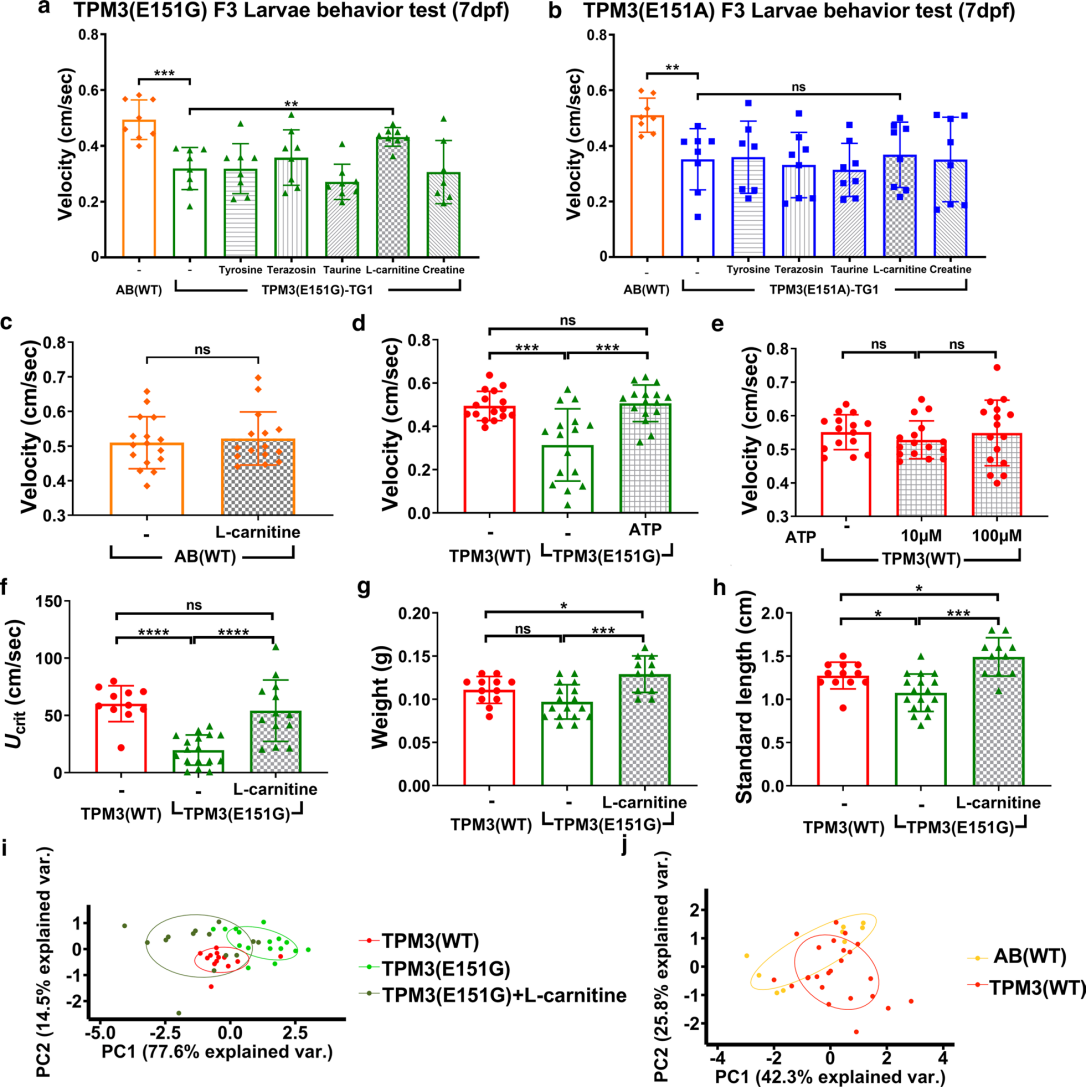
l-Carnitine treatment ameliorates swimming behavior of *TPM3*(E151G) larva and muscle endurance of *TPM3*(E151G) adult transgenic zebrafish. **a** F3 larvae *TPM3*(E151G) fish (*n* = 8) were treated with five different chemicals starting at 28 h-post-fertilization (hpf) for six days, and larvae swimming behavior was tested at 7 day-post-fertilization (dpf). l-Carnitine significantly increased the swimming velocity compared to un-treated control. **b** Five different chemicals were used to treat F3 *TPM3*(E151A) larvae (*n* = 8). l-Carnitine does not improve the swimming velocity for *TPM3*(E151A) transgenic fish. **c** AB(WT) larvae with and without l-carnitine treatment (*n* = 16). l-Carnitine has no effect on non-transgenic AB(WT) fish. **d** ATP treatment of F3 *TPM3*(E151G) larvae considerably improved the swimming speed. **e** ATP treatments on F3 *TPM3*(WT) larvae showed no improving effect. **f** F3 adult *TPM3*(E151G) fish (*n* = 8) were treated with l-carnitine starting at 28 hpf for one month, with a muscle endurance test conducted at 1 month of age in a swimming tunnel. **g** The body weight of the l-carnitine treated *TPM3*(E151G) transgenic fish. **h** The standard length of the L-carnitine treated *TPM3*(E151G) transgenic fish. **i** PCA of endurance test for adult F3 *TPM3*(WT) and *TPM3*(E151G) transgenic zebrafish after l-carnitine treatment. Red plot denotes *TPM3*(WT), green plot is *TPM3*(E151G), and dark green plot represents *TPM3*(E151G) transgenic zebrafish after l-carnitine treatment. Statistical significance was determined using a *t*-test, *0.01 < *P* ≤ 0.05; **0.001 < *P* ≤ 0.01; ***0.0001 < *P* ≤ 0.001; ****  ≤ 0.0001

Based on the results above, we found that *TPM3*(E151G) mutant zebrafish exhibited weaker muscle endurance and slower swimming behavior, resembling human patients. As no treatment exists for CM, we used the *TPM3*(E151G) zebrafish to screen for potential therapeutic drugs. *TPM3* transgenic fish (F3) were treated with five chemicals (l-carnitine, l-tyrosine, taurine, creatine, and terazosin) from 28 h-post-fertilization (hpf) to 7 day-post-fertilization (dpf), and we measured the swimming velocity by using DanioVision. Amazingly, we found that only l-carnitine significantly improved the swimming velocity of *TPM3*(E151G) larvae, but the other four chemicals did not (Fig. 4a). In addition, l-carnitine treatment is specific to *TPM3*(E151G) to improve the swimming velocity, since the swimming speeds of *TPM3*(E151A) (Fig. 4b) and AB(WT) (Fig. 4c) fish were not altered by l-carnitine treatment. The results indicate that l-carnitine specifically enhances the swimming velocity of *TPM3*(E151G) larvae, but not the swimming velocity of *TPM3*(E151A) and AB(WT) larvae.

l-Carnitine can store and regulate the supply of ATP to enhance muscle endurance [6]. Thus we examined whether supplementation with ATP can produce a similar effect. As shown in Fig. 4d, ATP supplementation also significantly increased the swimming velocity of *TPM3*(E151G) F3 larvae but had no beneficial effect to *TPM3*(WT) F3 larvae (Fig. 4e).

To confirm muscle endurance could be restored by l-carnitine, the *TPM3* transgenic fish (F3) were treated with l-carnitine from 28 hpf for 30 days and measured for the muscle endurance using a swimming tunnel at 30 dpf. As expected, the muscle endurance of *TPM3*(E151G) fish was restored by l-carnitine treatment (Fig. 4f). One month of l-carnitine treatment also increased the weight (Fig. 4g) and length of the *TPM3*(E151G) transgenic fish (Fig. 4h). Using principal component analysis (PCA), we observed that *TPM3*(WT) and *TPM3*(E151G) + l-carnitine overlapped, indicating l-carnitine effectively restored the *TPM3*(E151G) muscle endurance to resemble the muscle endurance of *TPM3*(WT) (Fig. 4i). However, one month of l-carnitine treatment on *TPM3*(E151G) transgenic zebrafish starting from the age of three months failed to restore the muscle endurance (Additional file 4: Fig. S2), implying the treatment must be applied early during muscle development. We applied PCA analysis for AB(WT) and *TPM3*(WT) for all the fish in this study, and found there is no difference between them (Fig. 4j). This data indicated that overexpression of wildtype *TPM3* does not contribute to pathology, including fish survival, swimming velocity, T-maze, and muscle endurance.

### Reductions of mitochondria basal respiration and ATP production and muscle pathology in *TPM3*(E151G) mutation transgenic fish can be rescued by l-carnitine treatment

**Fig. 5:**
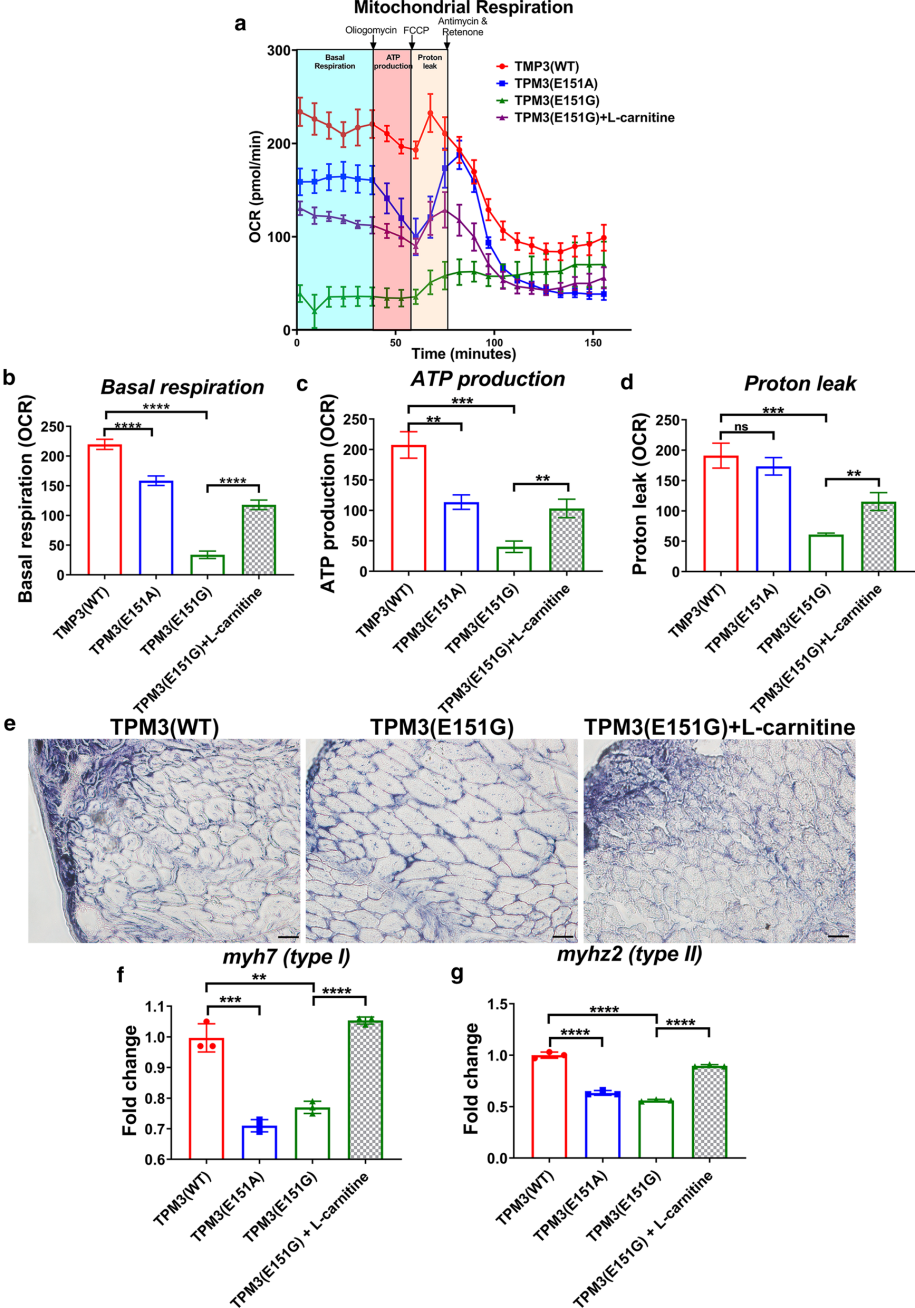
l-Carnitine treatment increased mitochndria activity and the type I and type II myosin light chain expression of *TPM3*(E151G) larva. Mitochondrial respiration of *TPM3* transgenic zebrafish via seahorse. **a** Schematic and oxygen consumption rate (OCR). OCR was measured under basal conditions followed by the sequential addition of oligomycin (0.5 μM), Carbonyl cyanide-4-(trifluoromethoxy) phenylhydrazone (FCCP) (4 μM), rotenone (1 μM), or antimycin A (1 μM). **b** Basal respiration, **c** ATP production, and **d** proton leak of OCR measurement. **e** NADH-TR stains of *TPM3*(WT), *TPM3*(E151G) and one month of L-carnitine treated *TPM3*(E151G) transgenic zebrafish at 400X, scale bar is 50 µm. **f** the expression level of *myh7* and **g** *myhz2* from *TPM3* transgenic fish (F3) treated with l-carnitine from 28 hpf to 7 dpf cpmpared to *TPM3*(WT). Statistical significance was determined using a *t*-test, *0.01 < *P* ≤ 0.05; **0.001 < *P* ≤ 0.01; ***0.0001 < *P* ≤ 0.001; ****P ≤ 0.0001

Since l-carnitine and ATP treatments both restored the muscle endurance of *TPM3*(E151G) transgenic fish, we wondered whether mitochondria respiration and ATP production were impaired in the *TPM3*(E151G) transgenic fish. The *TPM3*(WT), *TPM3*(E151A), *TPM3*(E151G), and l-carnitine-treated *TPM3*(E151G) transgenic fish were subjected to Seahorse XF Extracellular Flux Analyzer (Agilent Technologies, Santa Clara, CA, USA), and the mitochondria respiration data revealed *TPM3*(E151G) mutation caused significant decreases in basal respiration and ATP production (Fig. 5a–d).

To understand the muscle pathology changed after l-carnitine treatment, we performed NADH-TR stain for the one month of l-carnitine treated *TPM3* transgenic fish. Both the muscle volume and fiber size were increased in l-carnitine-treated *TPM3*(E151G) transgenic fish, and they were similar to *TPM3*(WT) (Fig. 5e). To further understand the muscle fiber type composition after l-carnitine treatment, we examined the expression of type I (*myh7*) and type II (*myhz2*) myosin fiber from *TPM3* transgenic fish (F3) treated with l-carnitine from 28 hpf to 7 dpf, and found that the expression level of *myh7* and *myhz2* in *TPM3*(E151A) and *TPM3*(E151G) was significantly reduced. After l-carnitine treatment, the expression of *myh7* and *myhz2* is significantly recovered to the same as *TPM3* (WT) (Fig. 5f, g). The effect of l-carnitine can also be found in both body weight and length. After feeding l-carnitine for one month, the weight and body length of *TPM3*(E151G) increased significantly indicated the fish became healthier (Additional file 5: Fig. S3).

### Next-generation sequencing discloses genes in ion channels and pathways dysregulated by *TPM3* mutation which can be reverted by l-carnitine treatment

To understand the molecular mechanism of *TPM3*-related CM, we extracted the RNA from the muscle specimens of adult *TPM3* mutant and WT transgenic fish and performed next-generation sequencing (NGS) analysis. Downregulation of genes in two *TPM3* mutant fish compared to *TPM3*(WT) were identified as involved in anatomy structure development, cell differentiation, and cell morphogenesis from the expression profiling of the muscle specimens (Additional file 6: Fig. S4A). Genes involved in anatomy structure development were also downregulated in muscle from embryos (Additional file 6: Fig. S4B). The fold changes from *TPM3*(E151G) were found to be much more lower than *TPM3*(E151A) in adults (Additional file 6: Fig. S4A), consistent with the recorded lower muscular endurance in *TPM3*(E151G). Comparing the differentially expressed genes (DEGs) from embryos and adults and verified with qRT-PCR, we found genes involved in anatomy structure development were both downregulated in embryos and adults (Additional file 6: Fig. S4C), and could be rescued by l-carnitine treatment (Additional file 6: Fig. S4D, Additional file 7: Fig. S5). Among them, *caldesmon 1b (cald1b)* exhibits actin binding activity and myosin heavy chain binding activity.

**Fig. 6:**
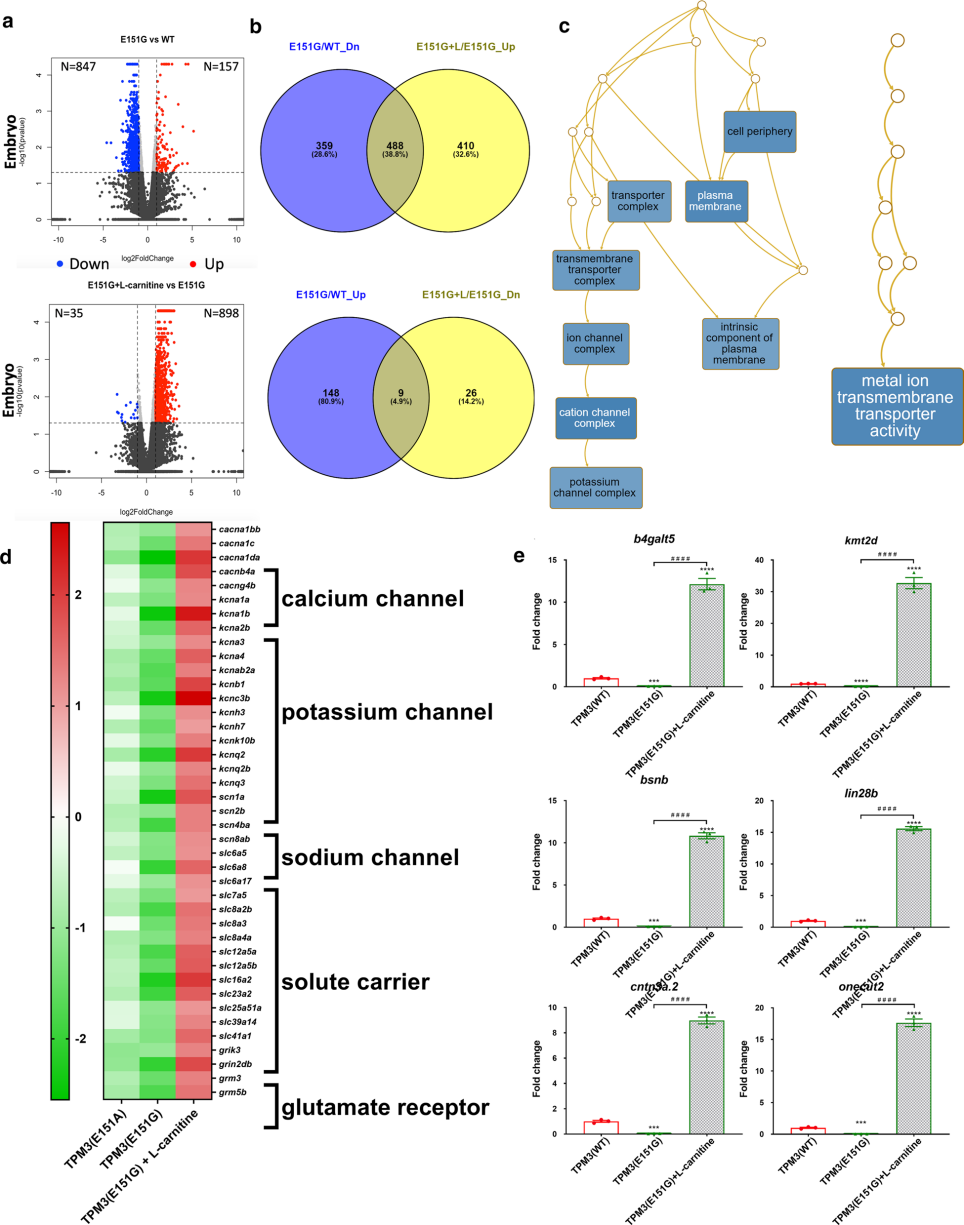
Deep sequencing data for *TPM3* transgenic zebrafish. **a** Volcano plot of genes differentially expressed between *TPM3*(E151G) mutant compared to *TPM3*(WT) (upper panel) and *TPM3*(E151G)-l-carnitine treatment compared to *TPM3*(E151G) (lower panel). **b** Venn diagram showing the overlap genes of downregulated in *TPM3*(E151G) compared to *TPM3*(WT) and upregulated in *TPM3*(E151G)-l-carnitine compared to no treatment *TPM3*(E151G) (upper panel) and overlap genes of upregulated in *TPM3*(E151G) compared to *TPM3*(WT) and downregulated in *TPM3*(E151G)-l-carnitine compared to no treatment *TPM3*(E151G) (lower panel). **c** Gene ontology analysis revealed the genes involved in metal ion transmembrane transporter activity are downregulated in *TPM3*(E151G) and reversed by l-carnitine treatment. **d** Unsupervised cluster analysis and heat-map including genes involved in calcium, potassium and sodium channel, solute carriers, glutamate receptor are downregulated in *TPM3*(E151A), reduced more in *TPM3*(E151G), and upregulated by l-carnitine treatment. The red and green indicate high and low expression of Log2 ratio, respectively. The results were obtained from next-generation deep sequencing. **e** The expression pattern of selected candidate genes of muscle specimens from embryos of *TPM3*(E151G) and l-carnitine-treated *TPM3*(E151G) compared with *TPM3*(WT) transgenic fish by qRT-PCR. Statistical significance was determined using a *t*-test, *0.01 < *P* ≤ 0.05; **0.001 < *P* ≤ 0.01; ***0.0001 < *P* ≤ 0.001; ****P ≤ 0.0001

We further analyzed the overlapped genes between *TPM3*(E151G) mutant and *TPM3*(E151G) with l-carnitine treatment, and found 488 overlapped genes which were downregulated more than two-fold in *TPM3*(E151G) and upregulated in *TPM3*(E151G) with l-carnitine treatment, but only 9 overlapped genes whose expressions were upregulated in *TPM3*(E151G) and downregulated in *TPM3*(E151G) with l-carnitine treatment (Fig. 6a,b). Using gene ontology analysis via WebGestalt [26], we found those overlapped genes downregulated in *TPM3*(E151G) and rescued by l-carnitine treatment involved in ion transmembrane transporter, especially potassium, calcium and sodium ion channels were enriched (Fig. 6c, d), the differentially expressed genes involved in anatomy structure development and ion channels were validated using qRT-PCR (Fig. 6e). Interestingly, sodium channel mutations has been reported to associate with severe myopathy with hypotonia hypokinesia or classical CM [28]. The results reveal some potential target genes in ion channels and pathways as the molecular mechanism of the *TPM3* mutant related CM, which may be used for further therapeutic targets.

### Ingenuity pathway analysis (IPA) revealed differential dysregulated pathways, functions and upstream regulators between *TPM*3(E151A) and *TPM3*(E151G)

**Fig. 7:**
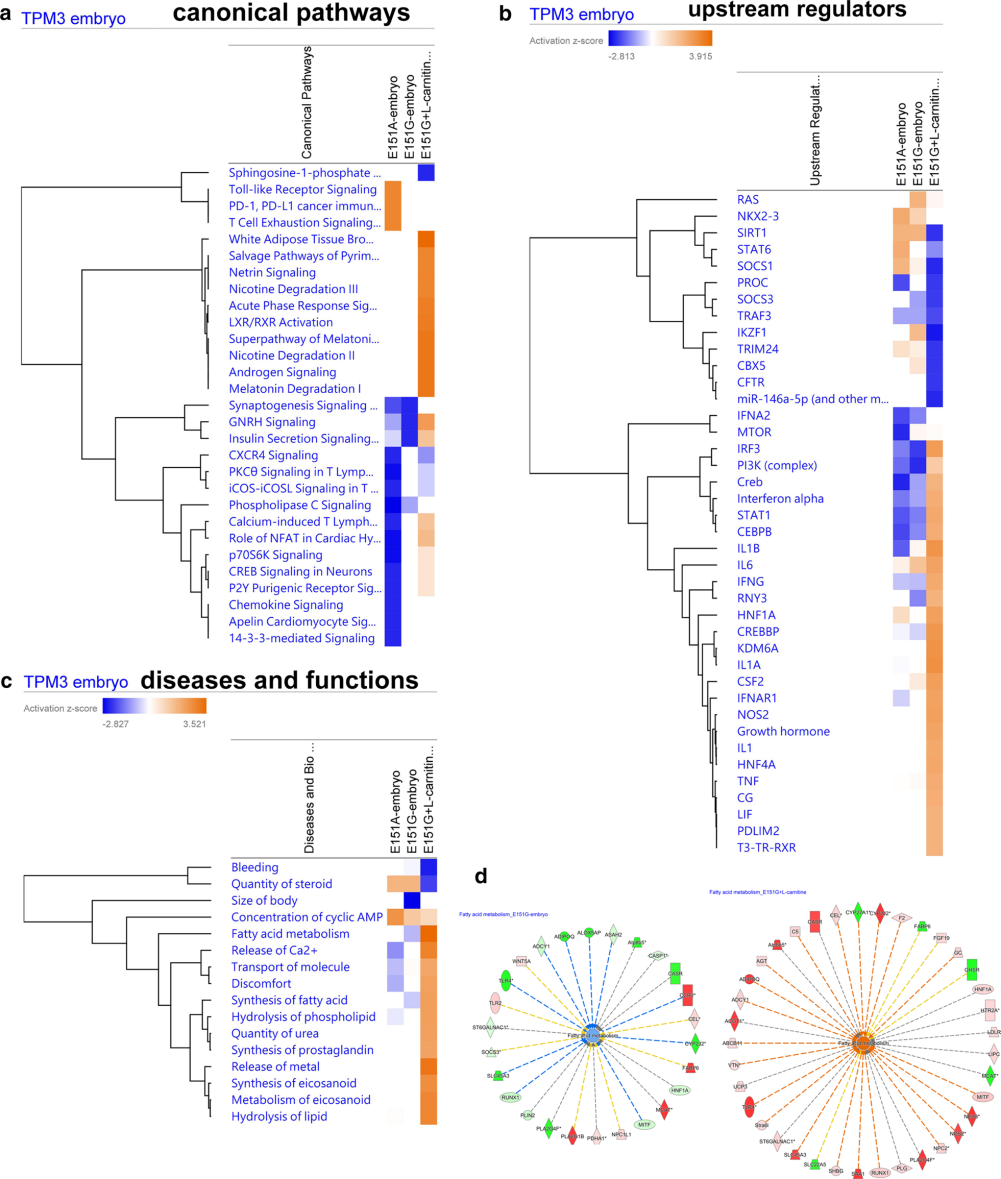
Analysis and interpretation the embryos of *TPM3*(E151A) and *TPM3*(E151G) transgenic fish with ingenuity pathway analysis. **a** List canonical signaling pathways and metabolic pathways affected by the *TPM3*(E151A), *TPM3*(E151G) and l-carnitine treated *TPM3*(E151G) by IPA analysis presented as hierarchical clustering. **b** List the upstream molecules related to the changing molecules in the *TPM3*(E151A), *TPM3*(E151G) and l-carnitine treated *TPM3*(E151G), and predict whether they are activated or inhibited based on the research literature presented as hierarchical clustering. **c** List of various analytical diseases and biological functions dysregulated by *TPM3*(E151A), *TPM3*(E151G) and l-carnitine treated *TPM3*(E151G) presented as hierarchical clustering. **d** Fatty acid metabolism is downregulated in *TPM3*(E151G) (left) and upregulated by l-carnitine treatment in *TPM3*(E151G) (right). The genes differential expressed are shown in red or blue indicated up-regulated or downregulated respectively. The blue or orange color in the center indicated fatty acid metabolism are downregulated or upregulated respectively

From pathological findings, muscle functional analyses and pharmacological responses, *TPM3*(E151A) and *TPM3*(E151G) show quite different phenotypes, from gene expression analysis, *TPM3*(E151A) and *TPM3*(E151G) also display differential gene expression, we have carry out Ingenuity Pathway Analysis (IPA) to find out the enriched pathways, diseases and functions as well as upstream regulators. In the embryos, *TPM3*(E151A) and *TPM3*(E151G) share few pathways, for example they both downregulated the synaptogenesis signaling pathways, GNRH signaling and insulin secretion signaling (Fig. 7). However, *TPM3*(E151A) dysregulated many different canonical pathways, upregulated Tol-like receptor signaling pathways and T cell exhaustions signaling pathways are only observed in *TPM3*(E151A) (Fig. 7a). In the upstream regulators, the *TPM3*(E151A) and *TPM3*(E151G) embryos share many similar upstream regulators, for example they both downregulated the *IFNA2*, *IRF3*, *PI3K*, *Creb*, *STAT1*, *CEBPB*, and l-carnitine treatment can reverse those changes (Fig. 7b). The biological changes among *TPM3*(E151A) and *TPM3*(E151G) are also different (Fig. 7c). E151G mutant downregulated the fatty acid metabolism and synthesis of fatty acid which can be reverse by l-carnitine treatment (Fig. 7d), but it was not seen in E151A. This can explain why the l-carnitine only can rescue the *TPM3*(E151G) but not *TPM3*(E151A) transgenic fish. However, release of Ca2 + , transport of molecule and hydrolysis of phospholipid are only downregulated in *TPM3*(E151A). Therefore, the *TPM3*(E151A) might need the Ca2 + transporter activator to rescue its muscle strength.

**Fig. 8:**
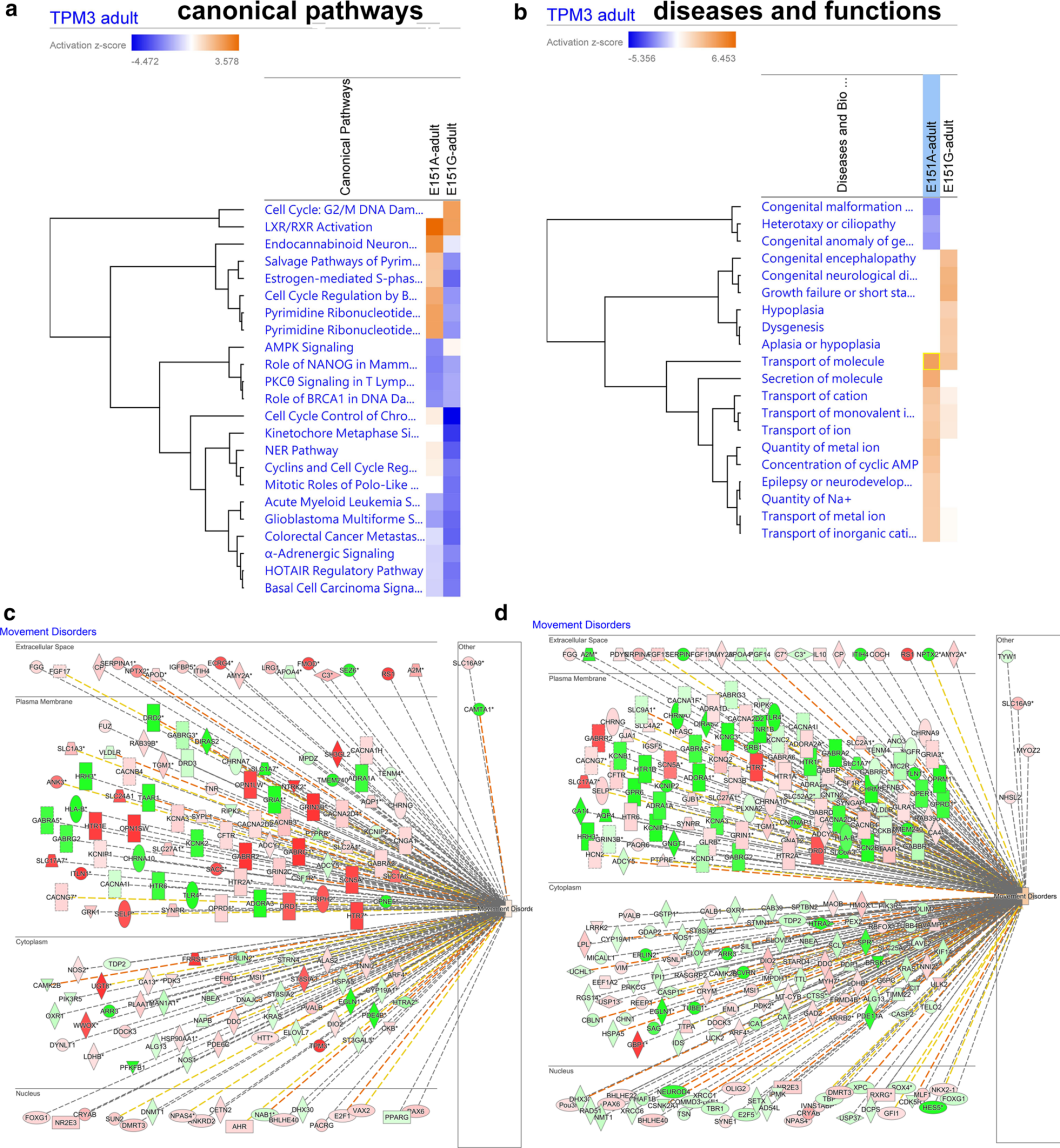
Ingenuity pathway analysis (IPA) network and canonical pathway analysis for the genes dysregulated in the *TPM3*(E151A) and *TPM3*(E151G) adult transgenic fish. **a** List canonical signaling pathways and metabolic pathways affected by the *TPM3*(E151A) and *TPM3*(E151G) adult by IPA analysis presented as hierarchical clustering. **b** List of various analytical diseases and biological functions dysregulated by *TPM3*(E151A) and *TPM3*(E151G) adult presented as hierarchical clustering. **c** Movement disorders is dysregulated in *TPM3*(E151A) adult fish presented as cellular compartment view. **d** Movement disorders is dysregulated in *TPM3*(E151G) adult fish. The genes differential expressed are shown in red or blue indicated up-regulated or downregulated respectively

The *TPM3*(E151A) and *TPM3*(E151G) adult share some pathways, but, *TPM3*(E151G) exhibited more severe downregulated canonical pathways than in *TPM3*(E151A) (Fig. 8a). The *TPM3*(E151A) and *TPM3*(E151G) adult share some biological functions, such as transport of molecule, transport of cation, transport of ion. However, the majority biological functions are differentially regulated in *TPM3*(E151G) and *TPM3*(E151A) (Fig. 8b). Both the genes’ expression dysregulated in *TPM3*(E151A) and *TPM3*(E151G)-adult involved in movement disorders (Fig. 8c, d).

## Discussion

In this study, we have generated transgenic zebrafish expressing wild type, E151A and E151G *TPM3* in muscles to study the pathogenicity of *TPM3*(E151G)-related CM. *TPM3*(E151G) fish displayed lower survival rate, severe skeletal abnormalities, and presented fiber type disproportion and exhibited weaker muscle endurance. Furthermore, we identified l-carnitine specifically improved the swimming velocity of *TPM3*(E151G) larvae and increased basal respiration and ATP production in mitochondria. The RNA-seq analysis from the muscles of *TPM3*(E151G) larvae revealed the downregulated genes involved in pathways of sodium, potassium and calcium ion channels which their downregulation can be reverted by l-carnitine treatment. *TPM3*(E151G) is a new novel mutation that has not been reported, and has much severe phenotypes than *TPM3*(E151A) mutation previously reported. Our study provides the evidence and prove that human *TPM3*(E151G) mutation was pathogenicity in *TPM3*-related CM by using zebrafish.

For the behavioral test, we started with the T-maze to measure the swimming velocity and memory test related to learning in our *TPM3* transgenic zebrafish. In ethology, the T-maze is a simple maze used for animal cognitive experiments. The T-maze is widely used to measure the spatial memory and learning tasks in laboratory animals, such as rat or zebrafish. Zebrafish has quickly become an important model for studying vertebrate neural development [29]. Due to its simple test for the function of learning, the T-maze has been widely used to study drugs and toxins that affect memory in zebrafish [30]. As in other studies, the *TPM3*(WT) zebrafish can be trained, indicating that *TPM3*(WT) zebrafish has demonstrated good learning ability and memory in the T-maze test. However, *TPM3* mutant zebrafish differed considerably from *TPM3*(WT), implying *TPM3* mutant zebrafish might experience memory deficits and are unable to learn. To eliminate the influence of learning ability, we used a swimming tunnel to test exact muscle endurance without learning ability. The swimming tunnel uses different water flowing velocities to keep the fish in a state of swimming to measure the muscle endurance. We proved *TPM3* mutation fish did reduce muscle endurance.

Using the *TPM3* mutant transgenic fish as a drug screening platform, we found l-carnitine specifically can restore the muscle endurance and swimming speed. Most of l-carnitine is intracellular and is stored in liver, skeletal muscle, heart, and kidney [31]. l-Carnitine promotes long chain fatty acyl CoAs into the mitochondria for β-oxidation and nonoxidative glucose disposal [32, 33]. Organic cation transporter-2 (OCTN2) enhances l-carnitine uptake inside cells. Carnitine acyltransferases are a family of enzymes that catalyze the reversible transfer of acyl groups between coenzyme A and l-carnitine and the conversion of acyl-CoA esters to acylcarnitine esters [34]. Neuroprotection was described in several animal models when administered with l-carnitine at super physiological concentrations. l-Carnitine was used to reduce lactate levels and elevate ATP levels upon administration [35]. A nemaline myopathy case with *ACTA-1* gene mutation and carnitine deficiency was the first described with evidence of a disorder of mitochondrial fatty acid oxidation [36].

The pathogenesis of CM is diverse. Based on recent emerging evidence, however, five key pathophysiological areas have been proposed, including defects in sarcolemmal and intracellular membrane remodeling and excitation–contraction coupling; mitochondrial distribution and function; myofibrillar force generation; atrophy; and autophagy [7]. We hypothesized that high concentrations of l-carnitine can enhance long chain fatty acyl-CoA into the mitochondria for β-oxidation to generate ATP to rescue muscle weakness.

In our *TPM3* transgenic zebrafish platform, we used larvae and adult fish for drug screening; some results from the larvae and adult fish platform seem unconcordant. We found that l-carnitine improve the swimming velocity of larval *TPM3*(E151G) zebrafish treated from 28 hpf for seven days (Fig. 4a). The muscle endurance of adult *TPM3*(E151G) zebrafish was rescued to near normal levels in adult treated l-carnitine from 28 hpf for one month (Fig. 4f), but l-carnitine could not rescue the muscle endurance of adult *TPM3*(E151G) zebrafish if treated at 3 months age (Additional file 4: Fig. S2A). It could be that adult fish muscle is well developed, the defect caused by *TPM3*(E151G) was unable to be rescued by l-carnitine if treated at 3 months of age. The myogenic regulatory factors, such as *Myod*, *Myf5*, and *Myogenin*, are expressed during the segmentation period [37, 38] at 1 day post fertilization during development [12], and decrease in adult stage. l-carnitine enhances long chain fatty acyl-CoAs into the mitochondria for β-oxidation to generate energy for skeletal muscle satellite (stem) cells to regenerate. The *TPM3*(E151G) larva zebrafish was rescued to near normal levels if treated l-carnitine from 28 hpf for seven days or one month. However, the stem cells are fewer with lower activity in adult of 3 month of age. Therefore, the muscle endurance cannot be improved in adult *TPM3*(E151G) zebrafish even if provided enough energy.

The sarcolemmal ion channels are crucial for muscle contraction involving excitation–contraction coupling machinery, sodium channelopathies are critical for myopathy, epilepsy disorders and complex encephalopathies [39]. Interestingly, sodium channel SCN4A mutations have been reported to be associated with severe fetal hypokinesia or classical CM [28]. In our study, we found genes involved in sodium channel: *scn1a*, *scn2*, *scn4ba* and *scn8ab* were upregulated by l-carnitine treatment (Fig. 6d). Calcium channel genes including calcium voltage-gated channel subunit alpha 1S (CACNA1S) [40], ryanodine receptor type 1 (RYR1) [41] and SH3 and cysteine rich domain 3 (STAC3) [42] and their mutations cause CM have been reported. In our study, we found calcium channel genes including: *cacna1bb, cacna1c, cacna1da, cacnb4a* and *cacng4b* were downregulated in *TPM3* mutation and their downregulation can be reverted by l-carnitine treatment (Fig. 6d). Mutations affects potassium channel has been reported to associate with myopathy in mouse [43]. In our study, we found potassium channel genes including: *kcna1a, kcna1b, kcna2b, kcna3, kcna4, kcnab2a, kcnb1, kcnc3b, kcnh3, kcnh7, kcnk10b, kcnq2, kcnq2b* and *kcnq3* were downregulated in *TPM3*(E151G) mutation and also can be reverted by l-carnitine treatment (Fig. 6d). The muscles of *TPM3*(E151G) larvae exhibits dysfunction of ion channels, l-carnitine treatment could reverse the gene expression of mainly sodium, potassium, and calcium channels, indicating that those ion channel genes may play key roles in pathophysiology of TPM3-related CM.

Although we found l-carnitine can improve the swimming velocity of larvae and the muscle endurance of *TPM3*(E151G) zebrafish if treated in the early stage for one month, treatment was not effective if started at three months of age. Clinically, human patients have often completed muscle development at the time of diagnosis, which is similar to adult fish. As with all CM, no FDA-approved treatment exists to date. Early implementation of NGS to enhance diagnostic capabilities of clinical suspected CM, especially overcome historic limitations of histopathologic and clinical overlap, must be developed so potential treatment can be administered as early as possible.

## Conclusions

Our transgenic fish serve as translational model of *TPM3 *de novo mutation for a clinically diagnosed boy who suffers from CM. It is the first study using transgenic zebrafish to prove the pathogenicity of a *TPM3*(E151G) novel mutation of CM. Moreover, we demonstrate that l-carnitine ameliorates the muscle endurance and pathophysiology of CM with *TPM3*(E151G) de novo mutation. This study highlights a role of potassium and calcium ion channels in *TPM3*-related CM and provides a potential mechanism of action of TPM3 pathophysiology.

## Supplementary Information

**Additional file 1: Table S1.** Primer information.

**Additional file 2: Figure S1.** The swimming velocity of larvae F1 *TPM3* transgenic zebrafish at 3–6 dpf.

**Additional file 3: Movie S1.** Swimming behavior of *TPM3*(E151A) and *TPM3*(E151G) compared with *TPM3*(WT) transgenic fish.

**Additional file 4: Figure S2.** L-carnitine treatment of F3 adult *TPM3* transgenic zebrafish starting at 3 months of age.

**Additional file 5: Figure S3.** L-carnitine treatment increased the body weight and length of *TPM3*(E151G) larva.

**Additional file 6: Figure S4.** A heatmap showing the deep sequencing data for *TPM3* transgenic zebrafish.

**Additional file 7: Figure S5.** The qRT-PCR validation of genes involved in anatomy structure development.

## Data Availability

The NGS data have been submitted to the NCBI Gene Expression Omnibus (GEO) under accession code GSE149261.

## Abbreviations

TPM3: Tropomyosin 3
CM: Congenital myopathy
CFTD: Congenital fiber type disproportion
AAALAC: Assessment and Accreditation of Laboratory Animal Care International
NHRI: National Health Research Institutes
MLC2: Myosin light chain 2
*U*_crit_: Critical swimming speed
OCR: Oxygen consumption rate
FCCP: Carbonyl cyanide 4-(trifluoromethoxy)phenylhydrazone
PCA: Principal component analysis
DMSO: Dimethyl sulfoxide
qRT-PCR: Quantitative real-time reverse transcription polymerase chain reaction
NADH-TR stain: Nicotinamide adenine dinucleotide dehydrogenase-tetrazolium reductase stain
HE stain: Hematoxylin and Eosin stain
cDNA: Complementary DNA
FPKM: Fragments per kilobase per million
KEGG: Kyoto encyclopedia of genes and genomes
TZCF: Taiwan Zebrafish Core Facility
ICOB: Institute of Cellular and Organismic Biology
dpf: Days post fertilization
MOHW: Ministry of Health and Welfare
