# Supplementary material for: l-Carnitine ameliorates congenital myopathy in a tropomyosin 3 de novo mutation transgenic zebrafish
Source: J Biomed Sci. 2021 Jan 12;28:8. doi: 10.1186/s12929-020-00707-1 (PMC7802209; doi:10.1186/s12929-020-00707-1)
Supplement: Supplementary file 1 — Additional file 1: Table S1. Primer information. [file 12929_2020_707_MOESM1_ESM.docx]

**Additional Table**

**Table S1.** Primer information.

| Primer Name | Sequence (5’ to 3’) |
| --- | --- |
| **For *TPM3* gene amplification** | |
| *TPM3*-F | ATGGCTGGGATCACCACC |
| *TPM3*-R | CTACATCTCATTCAGGTCAAGCAG |
| **For generated pME-*TPM3*** | |
| attB1-*TPM3*-F | GGGGACAAGTTTGTACAAAAAAGCAGGCTATGGCTGGGATCACCACC |
| attB2-*TPM3*-R | GGGGACAAGTTTGTACAAGAAAGCTGGGTCTACATCTCATTCAGGTCAAGCAG |
| **For confirming LR recombination reaction** | |
| MLC2-F2 | CCATCACTTTCCCCCTACCT |
| *TPM3*-R2 | CGGTCCAGCTCTTCTTCAAC |
| *TPM3*-F2 | AGCCAAGCTGGAAAAGACAA |
| p3E-polyAR | CCCCCTGAACCTGAAACATA |
| MLC2-F2 | CCATCACTTTCCCCCTACCT |
| p3E-polyAR | CCCCCTGAACCTGAAACATA |
| **For site-directed mutagenesis** | |
| *TPM3* A452S-F | CTGAGCTGGCAGSGTCCCGTTGCCG |
| *TPM3* A452S-R | CGGCAACGGGACSCTGCCAGCTCAG |
| *TPM3* A452G-F | CTGAGCTGGCAGGGTCCCGTTGCCG |
| *TPM3* A452G-R | CGGCAACGGGACCCTGCCAGCTCAG |
| **For confirming *TPM3* transgenic zebrafish** | |
| *TPM3*-NF | GATGAAGAAAAGATGGAACTCCAGG |
| *TPM3*-R | CTACATCTCATTCAGGTCAAGCAG |
| Q-myh7-F | TGTTCAACCTCAAAGAGCGC |
| Q-myh7-R | GGCTACAACCACCTCCTGAT |
| Q-myhz2-F | AGCTGTACGTCAAGGGAACA |
| Q-myhz2-R | GGGAGGATTCATTGGGTGGA |
| **For qRT-PCR confirming the adult NGS data** | |
| Q-cald1b-F | AGCCAAAGAGGAGGAAGAAAG |
| Q-cald1b-R | CACCCTTCGGAGCGATAAAT |
| Q-capn12-F | GAACAGTACCTGCCCTTCTATG |
| Q-capn12-R | TTCACCTCCTGGTTGGTTTC |
| Q-dock5-F | GGGACTACCTGAACAAATCCTC |
| Q-dock5-R | GTCGGGATCTTTCTCTCCTTTC |
| Q-rpgrip1l-F | CAGCATACACTCCTGCTGTAA |
| Q-rpgrip1l-R | CGTGGTCAGGAAAGTCATAGAG |
| Q-ush2a-F | CAGCCCTTCACGGAGTATTT |
| Q-ush2a-R | CACTTTGTGGAACTGTCTCTCT |
| Q-atp10a-F | TGGTCTGAGGACACTCTGTAT |
| Q-atp10a-R | CTCTCCTCTGTATGGCTGTTTC |
| Q-slc23a2-F | GAGGAATGGGTGTTGGGAAA |
| Q-slc23a2-R | AACCACAGGGATGGGATAGA |
| Q-slc24a4a-F | CTGGTGCTGATTGTCATGTATCT |
| Q-slc24a4a-R | GTGACCCTCTTGTCTTCCTTTC |
| **For qRT-PCR confirming the embryo NGS data** | |
| Q-abcc6a-F | ATAGCACACCGCCTCAATAC |
| Q-abcc6a-R | CTGGCCTCTCTTTGCTATAAGG |
| Q-arrb1-F | CGTCTACAAGAGCGACTGATAAA |
| Q-arrb1-R | CTTCTGGCCCTGGTTGTAAT |
| Q-cdh12a-F | GGTGAAGGAGTTGGCTCTATTT |
| Q-cdh12a-R | CTGCGCTCTCAGTGTGTAATAA |
| Q-cdh24a-F | CTCCAACTCTCCCATTCACTTC |
| Q-cdh24a-R | GGTGACAAGAGCACCAGATT |
| Q-cntnap1-F | GGCTGTGCTGTCAATCAAAC |
| Q-cntnap1-R | GGAATGACAGGTAGCCCTTATC |
| Q-kitb-F | CGGGATCCTATCGCTGTAATG |
| Q-kitb-R | TTCTCTCCTTCTGTGAGGTTAATG |
| Q-ntrk2b-F | GCAGCAGGAATGGTGTATCT |
| Q-ntrk2b-R | GGACATGCCAAAGTCTCCTATC |
| Q-shank1-F | CTGTCCACACACACCACTATC |
| Q-shank1-R | CCTCTCTCTTTCCCTCTCTCTT |
| Q-shank3a-F | CATCGAGGAGAAGAACGCTATG |
| Q-shank3a-R | CAGGTGTGGGTGTAAACTCTT |
| Q-si:ch73-334d15.2-F | AGCAGAACTGGTGTGTTATGG |
| Q-si:ch73-334d15.2-R | CCACCACATGGTTTGGGTAA |
| Q-slitrk5b-F | CGAACGTCTAACCGAGGATATG |
| Q-slitrk5b-R | AAAGTCCCAACCGCAATCT |
| Q-stat5-F | CTCCTCTGTGACTTCTCTCTCT |
| Q-stat5-R | TAAAGACCCACACCGAGAAAC |
| Q-zgc:114120-F | TAGGAGGGCGAGTGTGTTA |
| Q-zgc:114120-R | ACTCTGCCTGAGGATGTTTG |
| **For qRT-PCR to confirm the L-carnitine treatment** | |
| Q-SLC16A2-F | TTCCTGAAGATGGTGGGAAAG |
| Q-SLC16A2-R | GCCGATATGTCATCGACAAGA |
| Q-kmt2d-F | CAGGGTGTGGAGGAATCTTTAG |
| Q-kmt2d-R | GACCAGCTCTGGCTTCATATC |
| Q-lin28b-F | CGTGTTCGTTCACCAAAGTAAG |
| Q-lin28b-R | CAGACCTTTACTCGACCTCTTAAA |
| Q-nr6a1a-F | GATGCAGAGGAGAATGGGATAC |
| Q-nr6a1a-R | CAGCCCTCGCAGGAAATAA |
| Q-onecut2-F | GAAGACTGCCTATAACGCCTATC |
| Q-onecut2-R | CATGGCTTACTCCAGCATGTA |
| Q-b4galt5-F | GGGACACGCATACAGAAGAATA |
| Q-b4galt5-R | TGGAGAGCGAGAAGAGAAATAAC |
| Q-bsnb-F | TAGCAGGAGAGGCAATGTAAAG |
| Q-bsnb-R | GAGATGAGGTGGAAGAGGAATG |
| Q-cntn3a.2-F | CAGATGGTGTAACAGTGGAAGA |
| Q-cntn3a.2-R: | CGTCTTGGCCTGGATGATATAG |

**supplemental Figures**

**Fig. S1.** Expression level of *myh7* and *myhz2* in *TPM3* transgenic fish compared to AB(WT) and the swimming velocity of larvae F1 *TPM3* transgenic zebrafish at 3–6 dpf. (**A**) Expression level of *myh7* in *TPM3* transgenic fish compared to AB(WT). (**B**) Expression level of *myh7* in *TPM3* transgenic fish compared to AB(WT). Larvae fish swimming velocity was measured by DanoVision at (**C**) 3, (**D**) 4, (**E**) 5, and (**F**) 6 dpf of F1 *TPM3* transgenic larval zebrafish. All the statistics were calculated in comparison with AB(WT). Orange plot is AB(WT) (*n* = 12), red plot is the four TG lines of *TPM3*(WT) (*n* = 8 for each line), blue plot is the four TG lines of *TPM3*(E151A) (*n* = 8 for each line), and the green plot is the four TG lines of *TPM3*(E151G) (*n* = 8 for each line). Statistical significance was determined using a *t*-test, * 0.01 < *P* ≤ 0.05; ** 0.001 < *P* ≤ 0.01; *** 0.0001 < *P* ≤ 0.001; **** *P* ≤ 0.0001.

**Fig. S2.** L-carnitine treatment of F3 adult *TPM3* transgenic zebrafish starting at 3 months of age. (**A**) L-carnitine treated F3 adult *TPM3*(E151G) fish starting at 3 months of age for one month, and muscle endurance test at 4 months of age in a swim tunnel. (**B**) The body weight of the treated transgenic fish. (**C**) The standard length of treated transgenic fish. (**D**) PCA of endurance test for adult F3 *TPM3*(WT) and *TPM3*(E151G) transgenic zebrafish after L-carnitine treatment. Red plot denotes *TPM3*(WT), green plot is *TPM3*(E151G), and dark green plot represents *TPM3*(E151G) transgenic zebrafish after L-carnitine treatment. Statistical significance was determined using a *t*-test, * 0.01 < *P* ≤ 0.05; ** 0.001 < *P* ≤ 0.01; *** 0.0001 < *P* ≤ 0.001; **** *P* ≤ 0.0001.

**Fig. S3.** L-carnitine treatment increased the body weight and length of *TPM3*(E151G) larva. F3 larvae *TPM3*(E151G) fish were treated with L-carnitine at 28 hour-post-fertilization (hpf) for one month, and (**A**) The body weight (**C**) the standard length of the 8 groups were measured.

**Fig. S4.** A heatmap showing the deep sequencing data for *TPM3* transgenic zebrafish. (**A**) The expression pattern of candidate genes of muscle specimens from adult *TPM3* mutant compared to *TPM3*(WT) transgenic fish. (**B**) The expression pattern of candidate genes of muscle specimens from embryos of *TPM3* mutant compared to *TPM3*(WT) transgenic fish. (**C**) The deep sequencing data for selected differentially expressed genes from *TPM3*(E151G) adult and *TPM3*(E151G) embryos compared with *TPM3*(WT) transgenic fish. (**D**) The qRT-PCR verification of selected genes are shown. Statistical significance was determined using a *t*-test, * 0.01 < *P* ≤ 0.05; ** 0.001 < *P* ≤ 0.01; *** 0.0001 < *P* ≤ 0.001; **** *P* ≤ 0.0001.

**Fig. S5.** The qRT-PCR validation of genes involved in anatomy structure development. Those genes were downregulated in *TPM3*(E151A) and *TPM3*(E151G) compared with *TPM3*(WT) transgenic fish and rescued by L-carnitine treatment. Statistical significance was determined using a *t*-test, * 0.01 < *P* ≤ 0.05; ** 0.001 < *P* ≤ 0.01; *** 0.0001 < *P* ≤ 0.001; **** *P* ≤ 0.0001.
