# Supplementary figures and images for: l-Carnitine ameliorates congenital myopathy in a tropomyosin 3 de novo mutation transgenic zebrafish
Source: J Biomed Sci. 2021 Jan 12;28:8. doi: 10.1186/s12929-020-00707-1 (PMC7802209; doi:10.1186/s12929-020-00707-1)

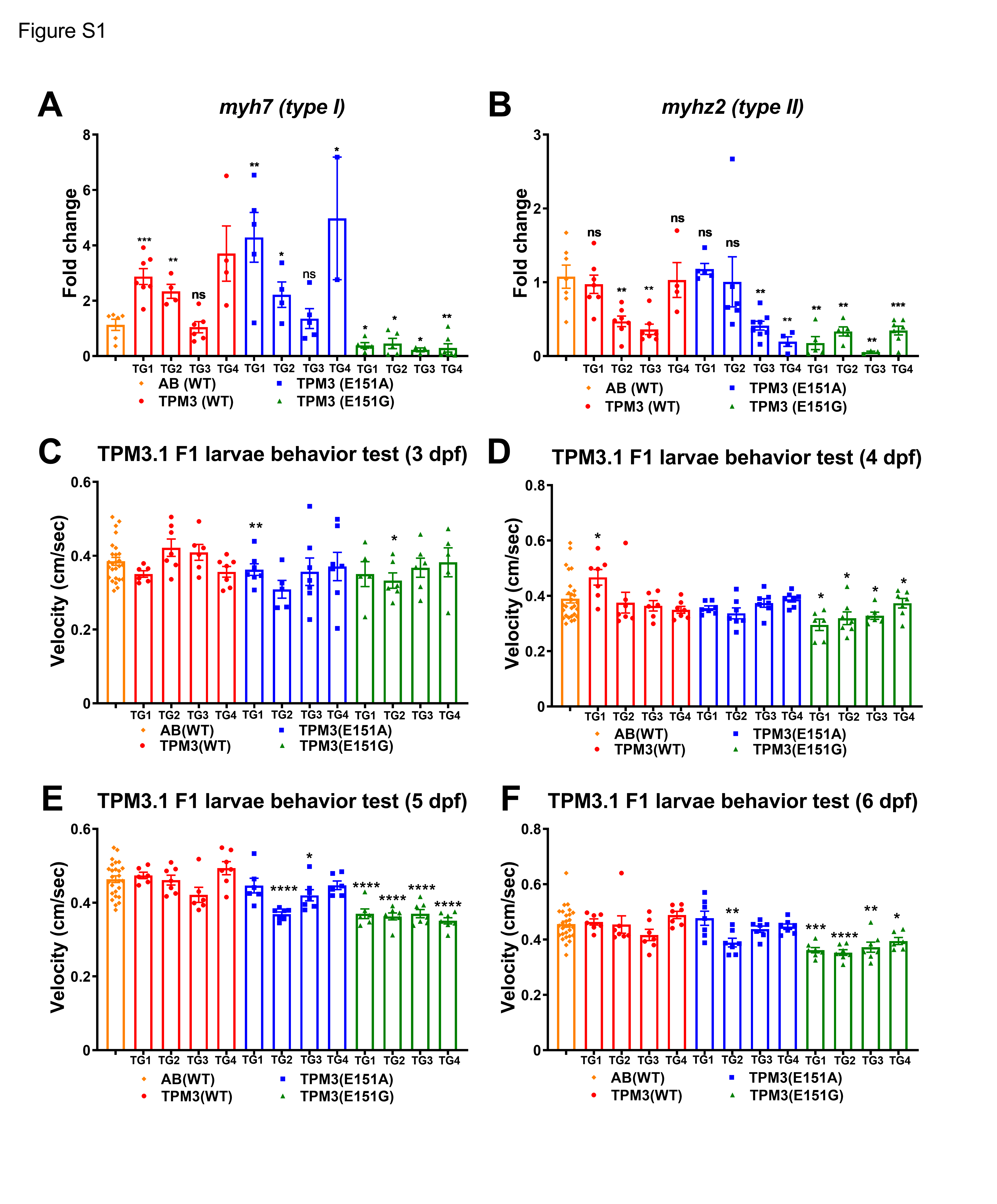

Supplement: Supplementary file 2 — Additional file 2: Figure S1. The swimming velocity of larvae F1 TPM3 transgenic zebrafish at 3–6 dpf. [file 12929_2020_707_MOESM2_ESM.tif]

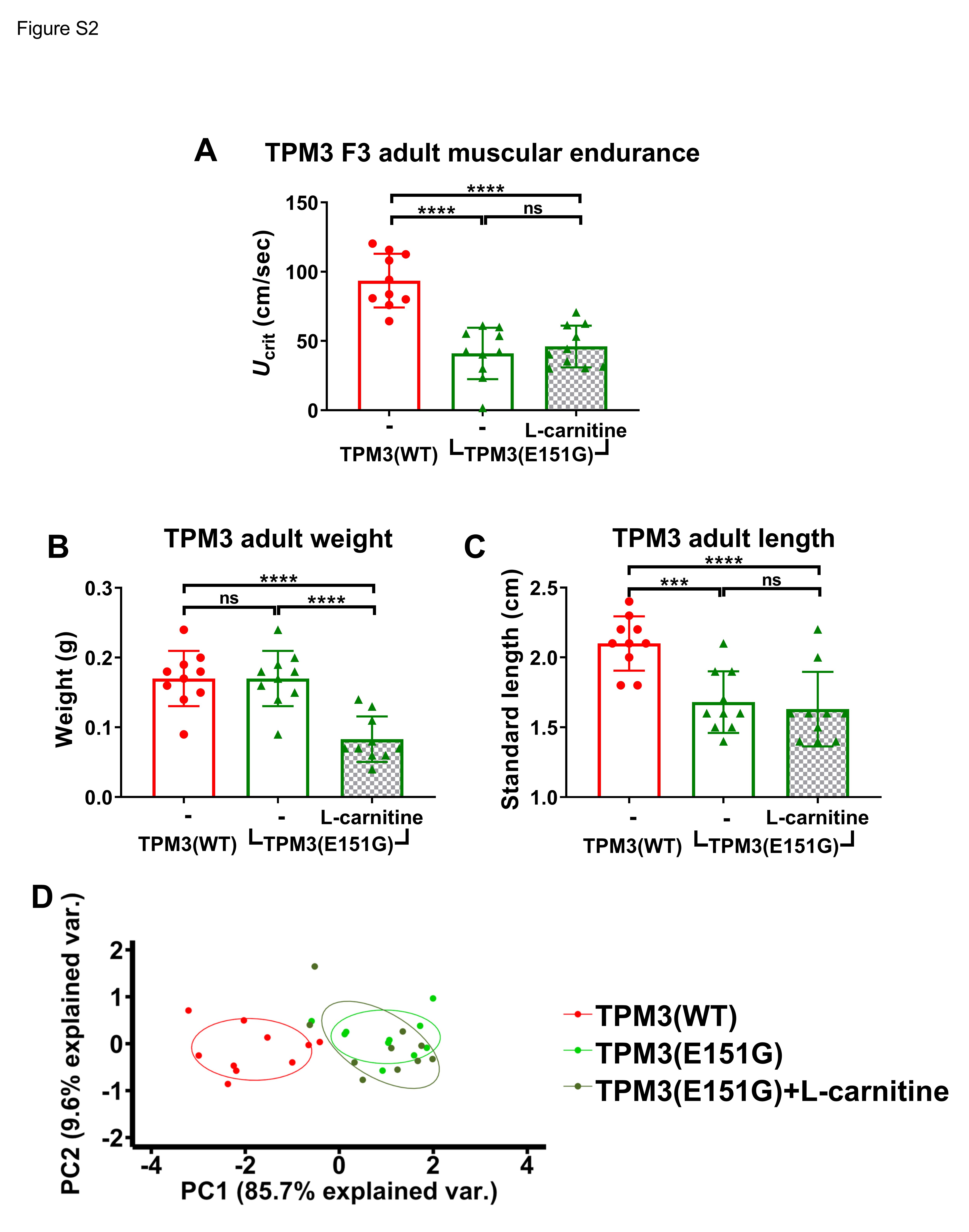

Supplement: Supplementary file 4 — Additional file 4: Figure S2. L-carnitine treatment of F3 adult TPM3 transgenic zebrafish starting at 3 months of age. [file 12929_2020_707_MOESM4_ESM.tif]

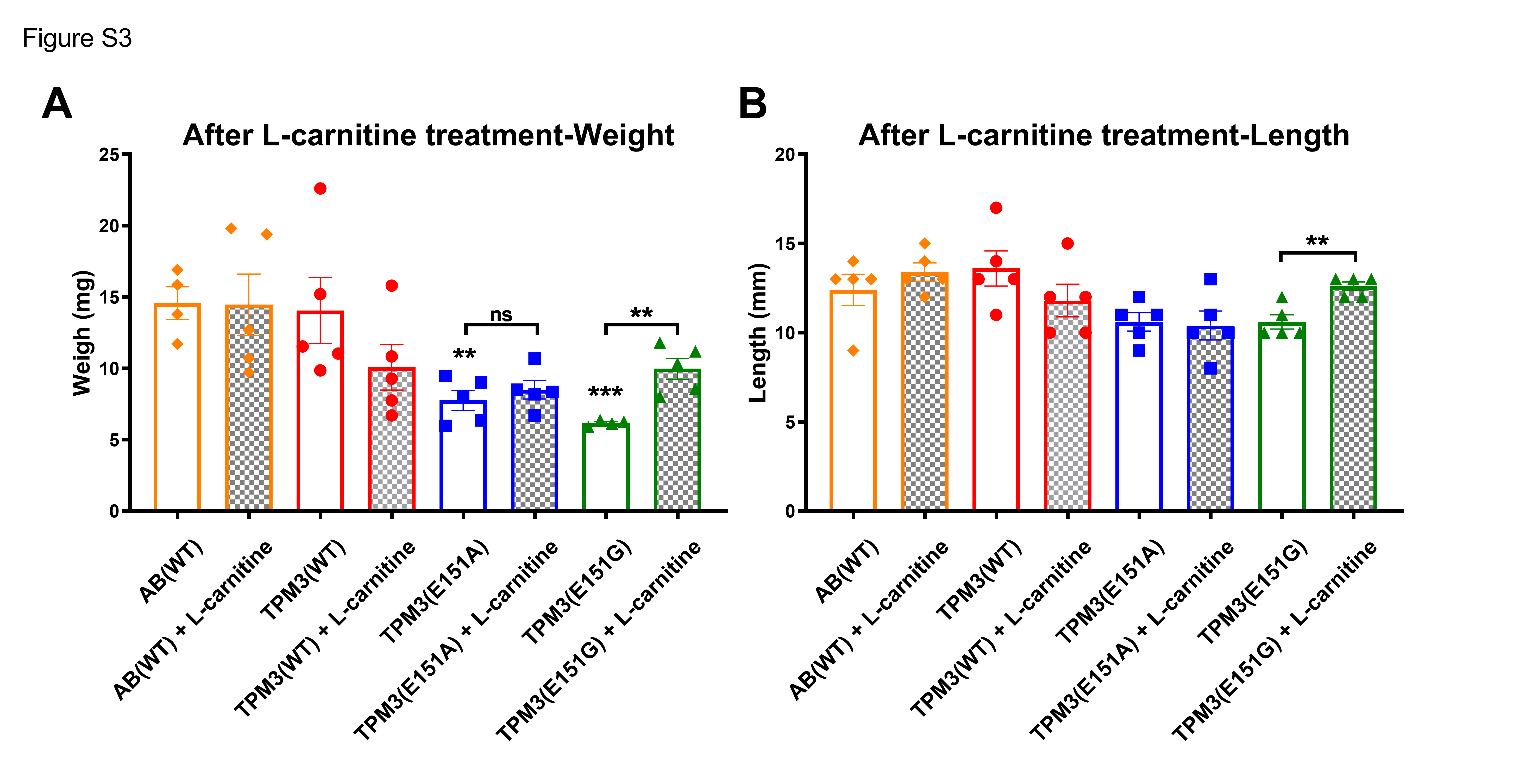

Supplement: Supplementary file 5 — Additional file 5: Figure S3. L-carnitine treatment increased the body weight and length of TPM3(E151G) larva. [file 12929_2020_707_MOESM5_ESM.tif]

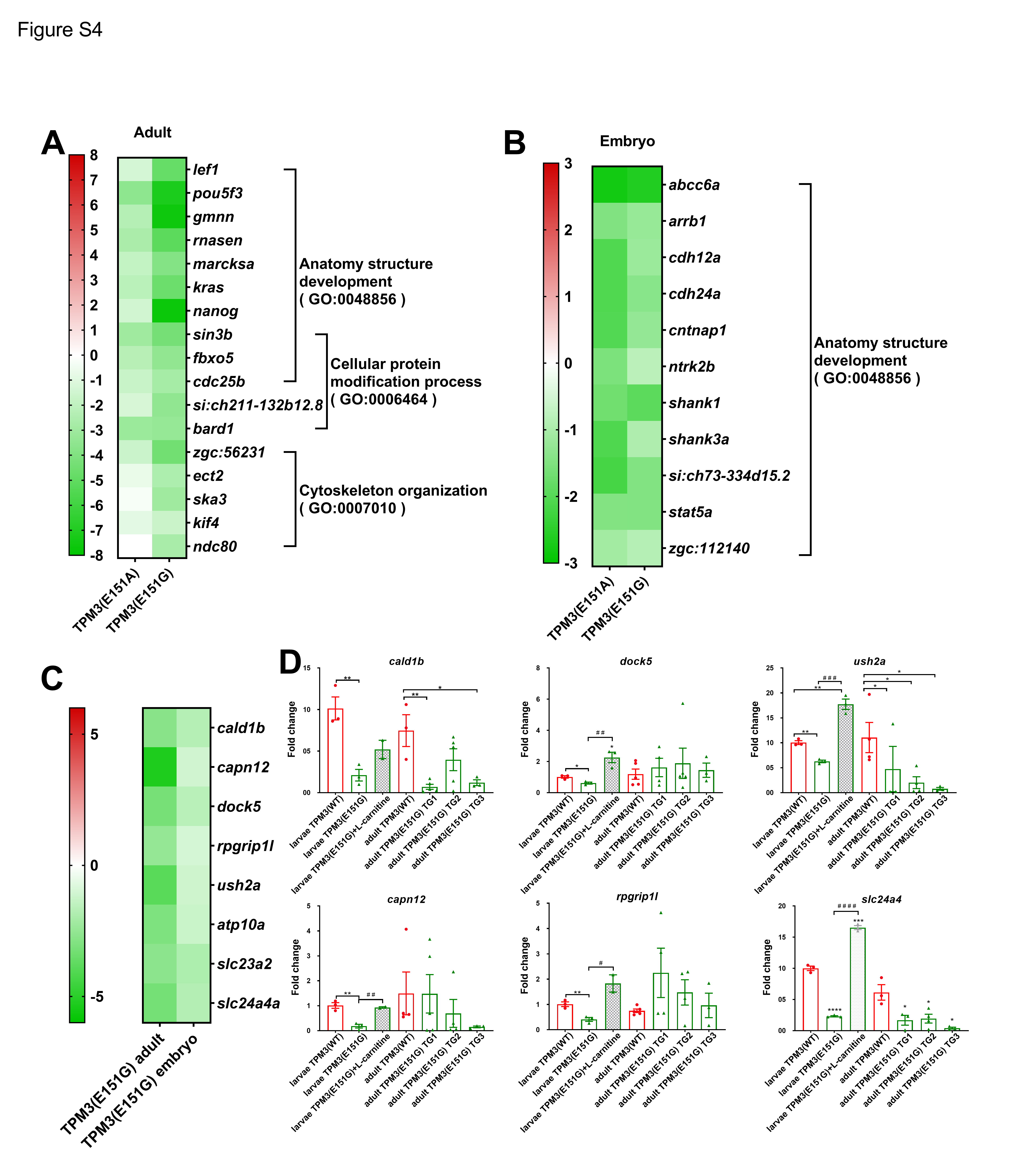

Supplement: Supplementary file 6 — Additional file 6: Figure S4. A heatmap showing the deep sequencing data for TPM3 transgenic zebrafish. [file 12929_2020_707_MOESM6_ESM.tif]

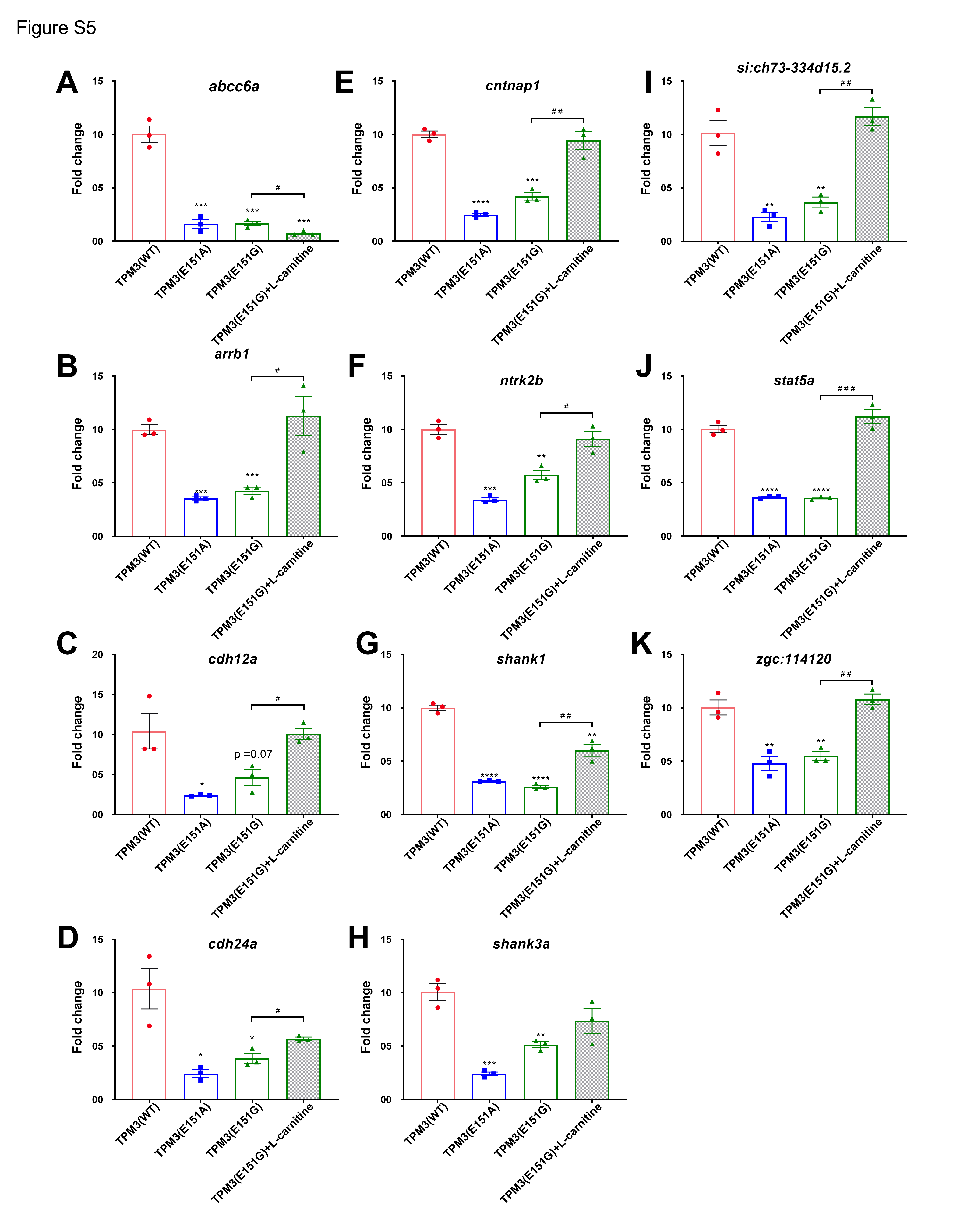

Supplement: Supplementary file 7 — Additional file 7: Figure S5. The qRT-PCR validation of genes involved in anatomy structure development. [file 12929_2020_707_MOESM7_ESM.tif]
